# Genome-Wide Inhibition of Pro-atherogenic Gene Expression by Multi-STAT Targeting Compounds as a Novel Treatment Strategy of CVDs

**DOI:** 10.3389/fimmu.2018.02141

**Published:** 2018-09-19

**Authors:** Martyna Plens-Galaska, Malgorzata Szelag, Aida Collado, Patrice Marques, Susana Vallejo, Mariella Ramos-González, Joanna Wesoly, María Jesus Sanz, Concepción Peiró, Hans A. R. Bluyssen

**Affiliations:** ^1^Department of Human Molecular Genetics, Institute of Molecular Biology and Biotechnology, Adam Mickiewicz University, Poznan, Poland; ^2^Department of Pharmacology, Faculty of Medicine, University of Valencia, Valencia, Spain; ^3^Institute of Health Research INCLIVA, University Clinic Hospital of Valencia, Valencia, Spain; ^4^Department of Pharmacology, School of Medicine, Universidad Autónoma de Madrid, Madrid, Spain; ^5^Instituto de Investigación Sanitaria Hospital Universitario La Paz (IdiPAZ), Madrid, Spain; ^6^Laboratory of High Throughput Technologies, Institute of Molecular Biology and Biotechnology, Faculty of Biology, Adam Mickiewicz University, Poznan, Poland

**Keywords:** vascular inflammation, STAT, *in silico* docking, multi-STAT inhibitors, CVDs treatment strategy

## Abstract

Cardiovascular diseases (CVDs), including atherosclerosis, are globally the leading cause of death. Key factors contributing to onset and progression of atherosclerosis include the pro-inflammatory cytokines Interferon (IFN)α and IFNγ and the Pattern Recognition Receptor (PRR) Toll-like receptor 4 (TLR4). Together, they trigger activation of Signal Transducer and Activator of Transcription (STAT)s. Searches for compounds targeting the pTyr-SH2 interaction area of STAT3, yielded many small molecules, including STATTIC and STX-0119. However, many of these inhibitors do not seem STAT3-specific. We hypothesized that multi-STAT-inhibitors that simultaneously block STAT1, STAT2, and STAT3 activity and pro-inflammatory target gene expression may be a promising strategy to treat CVDs. Using comparative *in silico* docking of multiple STAT-SH2 models on multi-million compound libraries, we identified the novel multi-STAT inhibitor, C01L_F03. This compound targets the SH2 domain of STAT1, STAT2, and STAT3 with the same affinity and simultaneously blocks their activity and expression of multiple STAT-target genes in HMECs in response to IFNα. The same *in silico* and *in vitro* multi-STAT inhibiting capacity was shown for STATTIC and STX-0119. Moreover, C01L_F03, STATTIC and STX-0119 were also able to affect genome-wide interactions between IFNγ and TLR4 by commonly inhibiting pro-inflammatory and pro-atherogenic gene expression directed by cooperative involvement of STATs with IRFs and/or NF-κB. Moreover, we observed that multi-STAT inhibitors could be used to inhibit IFNγ+LPS-induced HMECs migration, leukocyte adhesion to ECs as well as impairment of mesenteric artery contractility. Together, this implicates that application of a multi-STAT inhibitory strategy could provide great promise for the treatment of CVDs.

## Introduction

Cardiovascular diseases (CVDs) are globally the leading cause of death in Western Countries. Atherosclerosis is preceded by endothelial dysfunction, a prothrombotic and pro-inflammatory state of the endothelium which involves the increased expression of cell surface adhesion molecules, the production of inflammatory cytokines and chemokines and altered contractility of vascular smooth muscle cells (VSMCs) (1). Blood leukocytes are recruited to the injured vascular endothelium. This process is a hallmark of the initiation and progression of atherosclerosis. Recruitment of blood leukocytes involves many inflammatory mediators, modulated by cells of both innate and adaptive immunity (1). Pro-inflammatory cytokines Interferon (IFN)α, IFNγ and Toll-like receptor 4 (TLR4) activators are key factors contributing to early stages of atherosclerosis (2). IFNα and IFNγ induce phosphorylation of STATs through Janus-kinases (JAK)s. Thus, IFNα stimulates formation of STAT1 and STAT2 heterodimers, that complexed with IRF9 form ISGF3 and regulate expression of ISRE-containing genes. On the other hand, IFNα and IFNγ activate STAT1 or STAT3 homo-/heterodimer formation, which regulate expression of a distinct set of GAS-driven genes. IFNs also activate members of the IRF family including IRF1 and IRF8, that modulate a second wave of ISRE-dependent gene expression (3, 4).

Rapid activation of nuclear factor-κB (NF-κB) and IRFs is a result of TLR4 ligation (4–7). This leads to amplification of the initial inflammatory response, exertion of antimicrobial activities and initiation of acquired immunity. Several of the cytokines that are upregulated in the initial wave of immediate early gene expression e.g., IFNβ and TNFα, induce a secondary wave of STAT1 and STAT2 dependent gene expression and NF-κB signaling, respectively (4, 8, 9). On the other hand, IL-6 leads to the activation of STAT3.

IFNγ and TLR4 participate in signaling cross-talk through combinatorial actions of distinct and overlapping transcription factors on ISRE, GAS, ISRE/GAS, ISRE/NF-κB or GAS/NF-κB binding sites. As such, inflammation-induced activation of STAT1, STAT2, and STAT3, NF-κB and different IRFs coordinates robust expression of multiple chemokines, adhesion molecules, antiviral and antimicrobial proteins. Thus, signal integration between IFNγ and LPS in vascular cells and atheroma interacting immune cells modulates important aspects of inflammation, with STATs being important mediators (7, 10).

JAK-STAT pathway inhibitory strategies are numerous and one of the most promising is development of JAK inhibitors (Jakinibs), which exhibit the pan-JAK effect, defined as cross-binding to few JAKs e.g., FDA approved tofacitinib inhibits both Jak1 and Jak2. The concept of STAT inhibition is the more targeted approach, since STAT inhibitory strategies focus on affecting STAT dimerization. By exploring the pTyr-SH2 interaction area of STAT3, searches for STAT3-targeting compounds are numerous and yielded many small molecules, which can be called Statinibs (11, 12). Compared to Jakinibs these compounds affect expression of pro-inflammatory cytokines directly. Statinibs do not affect JAK-STAT signaling cascade upstream of the STAT phosphorylation and do not abrogate JAK action. Jakinibs might also influence, as a side effect, other JAK targets like SOCS or other kinases (e.g., Src and Abl). Of these STAT3-interacting compounds, STATTIC was shown to inhibit activation, dimerization, nuclear translocation of STAT3, and to increase apoptosis in STAT3-dependent cancer cell lines [reviewed in (7, 13)]. Similarly, the small-molecule STX-0119 was able to inhibit STAT3 dimerization and suppress human lymphoma SCC3 cell growth, through apoptosis and downregulation of known STAT3 targets. STX-0119 also exhibited potent antitumor effects *in vivo* of SCC3 tumor-bearing nude mice (14). Recently, we proposed a STAT cross-binding mechanism for STATTIC and STX-0119, in which both compounds target the SH2 domain of STAT1, STAT2, and STAT3 with similar affinity. We hypothesized that non-specific STAT-inhibitors, by simultaneous blocking STAT1, STAT2, and STAT3 activity (pan-STAT action) and expression of pro-inflammatory target genes, may be a promising avenue for the treatment of CVDs.

To prove this, we developed a pipeline approach which combines comparative *in silico* docking of multi-million CL and CDL libraries to multiple STAT-SH2 models with *in vitro* STAT inhibition validation, as a novel selection strategy for STAT-targeting inhibitors. This approach allowed us to identify a new type of multi-STAT inhibitor, C01L_F03 targeting the SH2 domain of STAT1, 2, and 3 with equal affinity. Moreover, we observed a similar STAT cross-binding mechanism for STATTIC and STX-0119, leading to genome-wide inhibition of pro-atherogenic gene expression directed by cooperative involvement of STATs with IRFs and/or NF-κB. Consequently, a multi-STAT inhibitory strategy was applied to inhibit endothelial cell (EC) migration, leukocyte adhesion to ECs and impairment of mesenteric artery contractility under inflammatory conditions.

## Materials and methods

### Protein model preparation

Three-dimensional models of STAT1, STAT2, and STAT3 were prepared based on the existing crystal structures for STATs deposited in RCSB Protein Data Bank: 1YVL, unphosphorylated STAT1 monomer; 1BF5, phosphorylated STAT1 dimer and 1BG1, phosphorylated STAT3 dimer [detailed description see Czerwoniec et al. (15) and Szelag et al. (16)]. Based on Chimera Dock Prep protocol AMBER ff99SB charges were applied to human STAT1, 2, and 3 models (17). Highly conserved pTyr-binding pocket (pY+0) and hydrophobic side-pocket (pY-X) in SH2 domain were selected for docking and virtual screening procedures. At the level of protein structures these SH2 domain superficial cavities are essential for STAT activation and binding of inhibitors (16, 18). To generate a “protomol,” “a pre-computed molecular representation of an idealized ligand” we used a ligand-based approach implemented in Surflex-Dock 2.6 (19). By definition protomol acts as the molecular probe of the active site to which ligands are matched (20). Ligand used to generate the protomol for STATs included a four amino acids from STAT-SH2 specific pTyr-linker matching the selected sub-pockets [STAT1, G**pY**^701^IK; STAT2, K**pY**^690^LK; STAT3, P**pY**^705^LK (15)].

### Compound library selection and small inhibitors preparation

Two small compound libraries of Clean Leads (CL) and Clean Drug-Like (CDL) were selected and downloaded from ZINC Database, with ready-to-dock parameters of protonation state and partial atomic charges (21). CL with molecular weight between 250 and 350 g/mol are smaller than most drugs. CDL chemical parameters fulfill criteria of the Lipinski‘s rule of five (22, 23). CL are in general more soluble than their bigger CDL cousins, and thus more likely to actually be assayed *in vitro*. In 2011 for the purpose of primary virtual screening (pre-screen) a CL subset has been downloaded, containing at that time 712,426 compounds. During the next step, similarity screening in 2013, CL subset in number of 4,591,276 and CDL subset in number of 13,195,609 compounds were selected.

Geometries of STAT3 inhibitors used for docking, STATTIC (24) and STX-0119 (25) were obtained from ZINC Database (code names ZINC00162014 and ZINC04107278 respectively). The structures were provided in ready-to-dock, 3D formats with molecules represented in biologically relevant forms (21).

### Virtual screening of small compound libraries

To select the top STAT1 inhibitors novel six-step virtual screening procedure was employed. The applied strategy is an antecedent to our more advanced protocol for big-scale virtual screening, named CAVS [see Czerwoniec et al. (15)]. The procedure used here is characterized by the following steps:

1. ***Pre-screen:*** For the CL library (712,426 compounds) docking simulations to STAT1 were carried out using Surflex-Dock 2.6 (19). Pscreen algorithm with fast screening parameter settings was employed (15, 20). For each compound we obtained 10 binding poses in the predefined area of the STAT1-SH2 domain. Additionally, each binding pose was supplied with a Binding Score value (BS) representing the total predicted binding affinity of the compound to the STAT1-SH2 domain. Moreover, input of polar interactions to the BS (represented by Polar Score) and the error rate of binding (represented by Crash) were also calculated.
2. ***Primary filtering of inhibitors:*** For each compound the best of ten binding poses was filtered out for further analysis. Then, we compared the binding quality between different compounds by using the STAT1-BS. Compounds with the highest STAT1-BS values were selected, checked for availability and 12 compounds (A01-L01) have been purchased for initial experimental validation.
3. ***Similarity screen:*** Based on the experimental results the best three compounds for STAT1 inhibition (C01, E01, and F01 from CL library) were used to perform a structural similarity screening. The CL list containing now 4,591,276 structures was screened with the criteria of at least 50% similarity to C01, E01, and F01. 1129 CL compounds fulfilled these criteria. Similarly, the CDL list with 13,195,609 structures was screened for compounds with >50% similarity and a molecular weight of ≥300 g/mol, to include bigger structures which could target a larger area of the SH2 domain. These criteria were fulfilled by 832 CDL compounds. Then for a total of 1,961 compounds, similarity scores (SIM and RMSD) were calculated using Surflex-Sim 2.6 (19) to assess the level of similarity to C01, E01, and F01.
4. ***Re-screen:*** Repeated docking simulations of a total of 1,961 compounds from the similarity screen to STAT1, 2 and 3-SH2 were carried employing the Surflex-Dock 2.6 pgeom algorithm which is recommended for detailed studies of relative alignments. More exhaustive parameter settings were used for optimal pose prediction of the compounds (15, 20). As a result, in the predefined area of STAT-SH2 domain we obtained 20 binding poses of each compound.
5. ***Secondary filtering of inhibitors:*** For each compound the best of 20 binding poses was filtered out for further analysis. Then by using the “Comparative Binding Affinity Value” for STAT1 [STAT1-CBAV, more details see Czerwoniec et al. (15)] the binding between STAT1, 2 and 3 was compared for each compound. Compounds with STAT1-CBAV≥0 were selected for graphical validation.
6. ***Binding diversity of conformers:*** Finally, graphical validation of the selected compounds was represented by the “ligand binding pose variation” (LBPV). This parameter reflects the docking accuracy. For detailed description of this procedure, see Czerwoniec et al. (15). The LBPV range of [0.8–1.0] represents low conformer diversity with very good binding specificity of the compound to STAT-SH2, whereas the range of [0.0–0.2] denotes high conformer diversity with poor binding specificity. Finally, compounds with the highest STAT1-CBAV and STAT1-LBPV values were selected, checked for availability and six compounds (C01L_A03 to C01L_F03) have been purchased for further experimental validation.

### Comparative docking of STATTIC, STX-0119, and C01L_F03

In order to compare STATTIC and STX-0119 with compounds obtained from CL and CDL virtual screening, docking simulations of STATTIC and STX-0119 to STAT1, 2 and 3 SH2 domain were performed with the pgeom algorithm (15, 20) implemented in Surflex-Dock 2.6 (19). For each structure in the predefined area of STAT-SH2 domain we obtained 20 binding poses. Then, for each compound the best of twenty binding poses was filtered out for further analysis. Finally, STAT1-CBAV was determined to compare the binding between STAT1, STAT2, and STAT3 for both compounds. Also, LBPV was used to validate the docking accuracy.

Moreover, we performed more exact docking simulations of STATTIC; two STATTIC analogs STB and STC; STX-0119; C01 and C01L_F03 for STAT1, 2 and 3 [see Szelag et al. (16)]. Geometries of two STATTIC analogs, which displayed lesser inhibition of STAT3 binding *in vitro* (13), were obtained from ZINC Database (code names ZINC00162015 and ZINC00162011 respectively). The structures were provided in ready-to-dock, 3D formats with molecules represented in biologically relevant forms (21). For all studied complexes of STAT1, 2 and 3 with STATTIC, STB, STC, STX-0119, C01, and C01L_F03 HADDOCK ligand docking protocol (26, 27) was used with addition of Surflex-Dock protocol (pgeomx algorithm) (16) to estimate the ΔG^0^ (free enthalpy change), which corresponds to the stability of the complex in a protein-ligand interaction in the equilibrium. More negative ΔG^0^ (higher free enthalpy change) corresponds to stronger interaction between ligand and the protein, which reflects better complex stability.

### *in silico* STAT-SH2 mutagenesis

We have performed docking studies of STATTIC, STX-0119 and C01L_F03 to wt and mutated STAT1, 2, 3 [with our STAT 3D models described in (16)]. For this purpose, HADDOCK ligand docking protocol (26, 27) was used with addition of Surflex-Dock protocol (16) to estimate the ΔG^0^ (free enthalpy change), which corresponds to the stability of the complex in a protein-ligand interaction in the equilibrium. We assumed that mutation of selected a.a. to alanine would impair binding of studied inhibitors to STAT1, 2, and 3-SH2 domain.

### Cell culture experiments

Recombinant IFNα and IFNγ were purchased from Merck, while LPS was provided by Sigma-Aldrich. C01 and C01L_A03-C01L_F03 were purchased from Enamine; E01 from Asinex; F01 from ChemDiv; STX-0119 from Merck and STATTIC from Sigma-Aldrich. Rabbit polyclonal antibodies against STAT1-pTyr^701^, tSTAT1, tSTAT2, tSTAT3 were obtained from Santa Cruz, STAT2-pTyr^689^ and STAT3-pTyr^705^ form Merck. Tubulin antibody was purchased from Merck and anti-rabbit HRP-conjugated antibody from Sigma-Aldrich. Human Microvascular Endothelial Cells (HMECs) (28) were provided by the Center for Disease Control and Prevention (Atlanta, GA) and cultured in MCDB-131 medium (IITD PAN, Wroclaw, Poland) containing 10% of fetal bovine serum (FBS) (Gibco, Thermo Fisher Scientific), 100 U/ml penicillin, 100 μg/ml streptomycin, 0.01 μg/ml EGF, 0.05 μM hydrocortisone and 2 mM L-glutamine. At least 12 h before the experiment, full medium was exchanged for serum starved-medium (containing 1% of FBS instead of 10%). After minimum 12 h-starvation HMECs were pre-treated with various concentrations of inhibitors: C01, E01, F01 (40 h) or C01L_F03 (48 h) or STATTIC (8 h) or STX-0119 (24 h). Additionally, HMECs were treated with 200 U/ml of IFNα (1 h for protein isolation or 4 h for RNA isolation), 10 ng/ml IFNγ (24 h or 8 h) and/or 1 μg/ml LPS (8 h or 4 h) or IL-6 (1 h) 100 ng/ml.

### Western blot analysis

HMECs were washed with phosphate buffered saline (PBS) and lysed using radio-immune precipitation assay (RIPA) lysis buffer (50 mM Tris-HCl, pH = 8.0, 150 mM NaCl, 1% Nonidet-40, 0.5% sodium deoxycholate, 0.1% SDS, 1% protein inhibitor cocktail, 1% EDTA, 0.1% PMSF) and stored at −80°C, as described in Sikorski et al. (4). Lysates were quantified using bicinchoninic acid (BCA) kit (Perce). Thirty micrograms of protein were loaded on Blot 4–12% Bis-Tris Plus Gels, electrophoresed and transferred to PVDF membranes (Santa Cruz). All western blot analyses were performed with Snap ID system (Merck). Membranes were blocked in 0.125% non-fat dry milk or 1% BSA in TBS-Tween (TBS-T) and incubated with primary antibodies (1:1000 pSTAT1, 1:500 tSTAT1, 1:500 pSTAT2, 1:500 tSTAT2, 1:3500 pSTAT3, 1:500 tSTAT3, 1:2000 tubulin) and then with secondary anti-rabbit HRP-conjugated antibody (1:2,0000). Immunoreactive bands were visualized by enhanced chemiluminescence (ECL) using Luminata Forte HRP Substrate (Merck) and detected with G:Box System (Syngene). After detection membranes were stripped with buffer containing 25 mM glycine, 1% SDS, pH = 2.0 and re-probed. The software Image Studio Lite from LI-COR Biosciences was used for western blot quantification with α-tubulin as reference protein.

### ChIP qPCR

ChIP experiments were performed as described by Daniel et al. in (29) with minor modifications (29). Briefly, 15 mln cells were seeded and pre-treated with 50 μM of C01L_F03 48 h and for 1 h with 200 U/ml of IFNα. Cross-linking with DSG (Sigma) was performed for 45 min and then with formaldehyde (Sigma) for 10 min. After fixation chromatin was sonicated with a Diagenode Bioruptor Plus to generate fragments with length of 200–1,000 bp. Chromatin was immunoprecipitated with antibodies against pSTAT1, pSTAT2, pSTAT3, and NF-κB p65 (Cell Signaling Technology®). Chromatin-antibody complexes were precipitated with anti-IgA and anti-IgG paramagnetic beads (Life Technologies). After four washing steps, complexes were eluted and the cross-links reversed. DNA fragments were column purified (Qiagen, MinElute). DNA was quantified with a Qubit fluorometer (Invitrogen). After immunoprecipitation DNA was quantified using quantitative PCR (qPCR) and normalized to values obtained after amplification of unprecipitated (input) DNA. Oligonucleotides sequences (Genomed) are in Table 1.

**Table 1:** List of primer sequences used in experimental procedures.

**Gene Name**	**Primer Sequence (5**^**′**^→**3**^**′**^**order)**	
	**Forward**	**Reverse**
ACTB	ACAGAGCCTCGCCTTTGCCGAT	ATCATCCATGGTGAGCTGGCGG
CXCL10	GCAGAGGAACCTCCAGTCTCAGCA	AGAGAGAGGTACTCCTTGAATGCCAC
CXCL9	GTGGTGTTCTTTTCCTCTTGGG	CTCACTACTGGGGTTCCTTGC
IFIT2	TCTTCCGTGTCTGTTCCATTC	AGCTGAAAGTTGCCATACCG
IRF1	GTCCAGCCGAGATGCTAAGAGC	TCTTCCGTGTCTGTTCCATTC
OAS2	CAATCAGCGAGGCCAGTAAT	TCCAGGTTGGGAGAAGTCAA
CCL5	CCATATTCCTCGGACACCAC	GGGTGACAAAGACGACTGCT
ICAM	CAGCGGCTGACGTGTGCAGTAA	TTGGGCGCCGGAAAGCTGTA
VCAM	TCCAGGTGGAGCTCTACTCATTCCC	TCCCATTCACGAGGCCACCACT
OAS2_ChIP	CGCTGCAGTGGGTGGAGAGA	GCCGGCAAGACAGTGAATGG
SOCS3_ChIP	CCATTCGGGAGTTCCTGGAC	TTGGCTTCTTGTGCTTGTGC
IRF1_ChIP STAT1	CCAAACACTTAGCGGGATTC	GAAATGACGGCACGCAG
IER3_ChIP	CCACCACCAGACTTCATCCC	GGAACTGCGGCAAAGTAGGA
IRF1_ChIP NF-κB	CTCAACAGCCAAGTGTGACC	GGCCAGCTTTACACCACAAG

### RNA isolation and quantitative reverse transcription-PCR (qRT-PCR) analysis

Total RNA was isolated using GeneMATRIX Universal RNA Purification Kit (EURx, Gdansk, Poland). 500 ng of total RNA was subjected to reverse transcription and PCR amplification was performed in Maxima SYBR Green/ROX qRT-PCR Master Mix (Thermo Fisher Scientific) on the Eco qRT-PCR System (Illumina). The amount of target gene in each sample was normalized to β-actin (ACTB) endogenous control (ΔCT). Data were transformed as described previously (30). Forward and reverse primers used in experiments are depicted in Table 1.

### Microarray analysis

Firstly, before treatment HMECs were starved for 12 h in 1% MCDB medium (IITD PAN, Wroclaw, Poland). Then cells were incubated with C01L_F03 (50 μM, 48 h) or STX-0119 (25 μM, 24 h) or STATTIC (10 μM, 8 h), IFNγ (10 ng/ml, 8 h) and LPS (1 μg/ml, 4 h) before RNA isolation. RNA was isolated from harvested cells with GeneMATRIX Universal RNA Purification Kit (EURx, Gdansk, Poland) and then labeled with Illumina®TotalPrep™ RNA Amplification Kit (Thermo Fisher Scientific). To obtain raw data Standard Illumina Expression BeadChip HumanHT-12v4 hybridization protocol was used. To avoid false results in case of all negative signals their value was changed to one, then signals were log-transformed. For further analysis, statistically significant average gene expression signals from two independent biological repeats were taken for statistical testing (GEO accession: GSE101508). Background subtraction and quantile normalization were applied and genes significantly (*p*-value ≤ 0.05) up-regulated at least 2-fold in any sample were selected for further analysis. IFNγ+LPS responsive genes that were commonly inhibited by C01L_F03, STATTIC or STX-0119 were selected according to the following formula: Fold Change (FC)_IFNγ+*LPS*_/FC_IFNγ+*LPS*+*inhibitor*_ value ≥4. Lists of inhibited genes were compared by Venn diagram analysis in the VennDiagram package in R (31). Identification of overlapping genes was based on “Gene ID and name.”

### Gene ontology enrichment analysis

Two datasets from microarray analysis (IFNγ+LPS induced genes; 731 in total; IFNγ+LPS responsive genes commonly inhibited by C01L_F03, STATTIC, and STX-0119) were mapped to gene ontology terms of biological process category using GOrilla webserver (32, 33). A *p*-value of 10^−3^ was used as a threshold and Illumina gene list from HumanHT-12v4 served as a background model. Then all statistically significant enriched GO categories were analyzed by REVIGO webserver (34) with medium similarity (0.7) and SimRel semantic similarity measure and mapped to *Homo sapiens* background to generate lists without redundant GO terms. Finally, the top 12 enriched GO terms with the highest fold enrichment for cells stimulated with IFNγ+LPS were selected and compared to those treated with tested compounds in presence of IFNγ+LPS.

### Promoter analysis

The initial list of 731 IFNγ+LPS induced genes was used for promoter analysis. The list was uploaded into pSCAN webserver (35) in search for ISRE, GAS and NF-κB binding sites. We analyzed 950 bp upstream and 50 bp downstream of the transcription start sites and obtained lists of overrepresented transcription factor binding sites, including matrix similarity score. Based on the results produced by pSCAN we chose matrices for further analysis. For checking distribution of: ISRE sequence we chose matrices: MA0652.1, MA0137.1, MA0.517.1; for GAS sequence: MA0137.2 and MA0137.3 and for NF-κB binding site: MA0105.1, MA0105.3. To prevent false positive results, we introduced threshold of matrix similarity score ≥ 0.85 for potential GAS, ISRE and ≥ 0.90 for potential NF-κB binding sites. To confirm “STAT specificity” of tested inhibitors, produced gene lists were merged for each individual binding site and compared with gene list of 159 genes inhibited commonly by C01L_F03, STATTIC and STX-0119 [by Venn diagram analysis by VennDiagram package in R (31)]. Identification of overlapping genes was based on “Gene ID and name.” The next step was to check if identified sequences may appear in one gene simultaneously. For that purpose, lists of genes containing either ISRE, GAS, NF-κB binding site were compared by Venn diagram analysis according to previously used protocol.

### *in vitro* wound healing assay

HMEC cells were split on 100 mm dishes and plated to reach high confluency. Cells then were starved in MCDB medium (IITD PAN, Wroclaw, Poland) with 0.1% of FBS for 12 h. Next step was to treat 2 dishes with 25 μM of C01L_F03 and 2 dishes with 25 μM of STX-0119. After 12 h of pre-incubation with C01L_F03 or STX-0119 scratches in these dishes were made. Another set of 2 dishes was treated with 10 μM of STATTIC 12 h before pictures were taken. At the same time 10 ng/ml of IFNγ and 1 μg/ml of LPS were added to one dish from each pair treated with C01L_F03, STX-0119 or STATTIC. Additionally, scratches were also made in set of 2 dishes that remained not treated with any inhibitor, one was used as an untreated control and to the second only IFNγ and LPS were added. Pictures were taken with Axio Observer.Z1 Microscope (Zeiss) after 12 h since the moment when scratches were made. The images acquired for each sample from two independent repeats were further analyzed quantitatively by ImageJ (36). For each image, 20 distances between one side of scratch and the other were measured at certain intervals (μm). By comparing the images from or inhibitor (with or without IFNγ+LPS treatment) to control, the distances of each scratch closure were obtained. Analysis of HMECs migration according to wound healing *in vitro*, which was performed according to Liang et al. (37).

### Leukocyte-endothelial cell interactions under flow conditions

Human umbilical vein endothelial cells (HUVECs) were isolated by collagenase treatment (38, 39) and maintained in human endothelial cell specific medium (EBM-2, Lonza, Verviers, Belgium), supplemented with endothelial growth media (EGM-2, Lonza) and 10% fetal bovine serum (FBS, Lonza). Cells up to passage 1 were grown to confluence to preserve endothelial features. Cells were incubated for 24 h in medium containing 1% FBS prior to every experiment.

Mononuclear cells were obtained from buffy coats of healthy donors by Ficoll Hypaque density gradient centrifugation (39, 40). The Glycotech flow chamber was assembled and placed on an inverted microscope stage. Freshly isolated mononuclear cells (1 × 10^6^/ml) were then perfused across the endothelial monolayers (HUVECs) unstimulated or stimulated with IFNγ (10 ng/ml, PreproTech, London, UK) for 24 h and LPS (1 μg/ml, Sigma Aldrich, Madrid, Spain) for 4 h. In the experiments cells were incubated with STATTIC 5 μM for 4 h or 1 μM for 24 h, STX-0119 25 μM or C01L_F03 50 μM for 24 h. In all experiments, leukocyte interactions were determined after 5 min at 0.5 dyn/cm^2^. Cells interacting with the surface of the endothelium were visualized and recorded (× 20 objective, × 10 eyepiece) using phase-contrast microscopy (Axio Observer A1 Carl Zeiss microscope, Thornwood, NY) (41).

### *Ex vivo* contractility studies

Four-month-old male C57Bl/6 mice were used for vascular reactivity experiments. Animals were maintained under standardized conditions with an artificial 12 h dark-light cycle, with free access to food and water. All animal studies were performed according to national guidelines and approved by the institutional animal care committees of Spain.

Immediately following sacrifice, the mesentery was removed, and placed in a Petri dish containing Krebs-Henseleit solution (KHS) at 4°C. The first branch mesenteric arteries (mean internal diameter ranged between 200 and 250 μm with non-significant differences observed among the different groups of mice) were dissected and mounted as ring preparations on a small-vessel myograph (DMT, Aarhus, Denmark) to measure isometric tension (42). The microvessels were exposed to 125 mM KCl to achieve a stable contraction, after which they were washed three times with KHS and a further 30 min. washout period was allowed. At this point, the vascular segments were maintained for 4 h prior to the exposure to increasing concentrations of noradrenaline (NA; 10^−10^ to 10^−6^M) to assess vascular contraction. In some experiments, the vascular segments were exposed to STATTIC (1 nM), STX-0119 (10 nM) or C01L_F03 (1 μM), IFNγ (10 ng/ml for 3 h prior to NA stimulation), and/or LPS (1 μg/ml for 1.5 h prior to NA stimulation) based on a previous report (10). Because of incubation time limitations of the system (< 8 h), we were able to test STATTIC, C01L_F03 and STX-0119 only for 4 h prior to NA stimulation.

### Statistical analysis

Results of qRT-PCR assay are presented as mean ± SEM for three independent repeats. Results of wound healing assay are presented as mean ± SEM for two independent repeats. Data for both experiments were compared by two-way ANOVA and unpaired two-tailed student *T*-test as indicated. A probability value *p* < 0.0001 was considered statistically significant. Results of mononuclear cell adhesion to HUVEC assay are presented as mean ± SEM for five to seven independent repeats. Data were compared by one-way ANOVA and unpaired two-tailed student *T*-test. A probability value p < 0.05 was considered statistically significant. Results of *ex vivo* contractility studies are presented as mean ± SEM for six to eighteen independent repeats. Data were compared by two-way ANOVA. A probability value *p* < 0.05 was considered statistically significant. All statistical tests were performed with GraphPad Prism version 7.0a for Mac OS X, GraphPad Software, La Jolla California USA, www.graphpad.com.

## Results

### Identification of C01, E01 and F01 as novel low potent STAT1-SH2 inhibitory compounds

**Figure 1:**
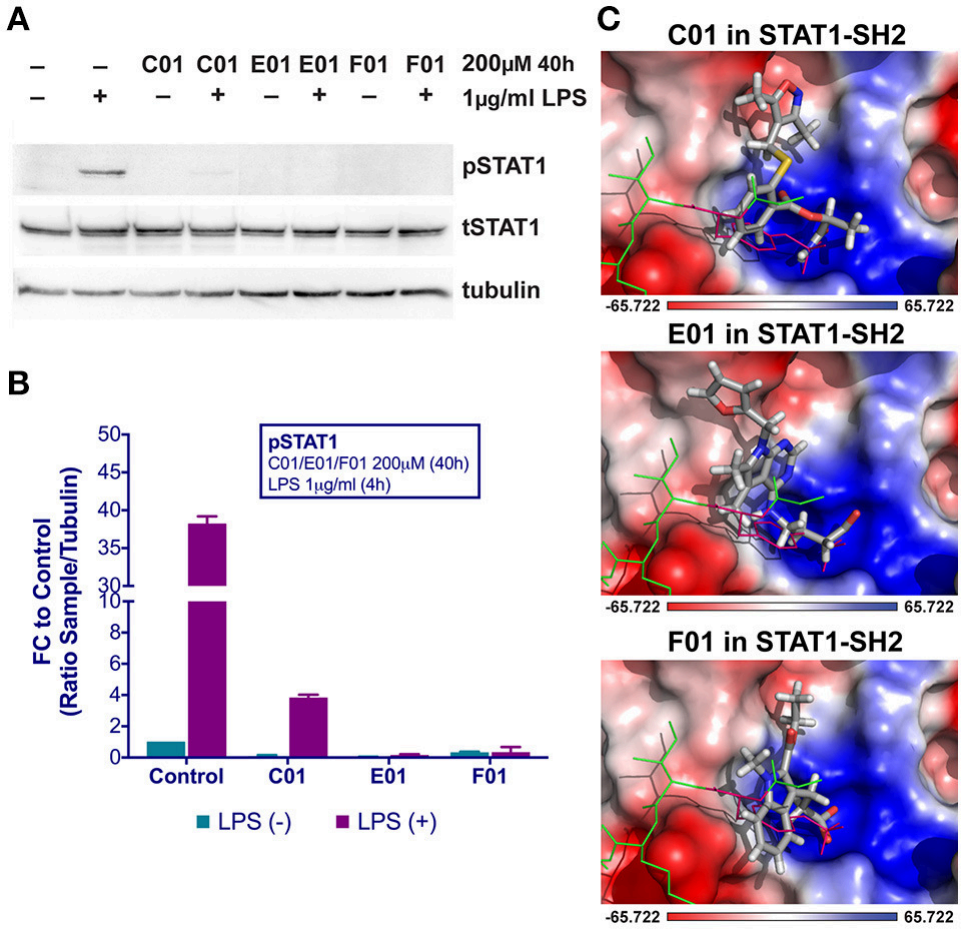
C01, E01, and F01 inhibit LPS-induced STAT1 phosphorylation **(A)**. HMECs were treated with 200 μM of tested compounds for 40 h and with 1 μg/ml of LPS for 4 h. Protein extracts were collected and levels of pSTAT1, tSTAT1 and α-tubulin were assessed by western blotting. Western quantification **(B)**. Bars represent mean quantification form 3 individual repeats ± SEM as error bars. Top-scored binding conformations of C01, E01, and F01 in the SH2 domain of STAT1 **(C)**. C01, E01, and F01 compounds are shown in stick representation and colored according to the atomic structure. pTyr-linker is represented by green lines with pink pTyr residue. SH2 domain of STAT1 in the surface representation is colored based on the distribution of the APBS electrostatic surface potential (43). Positively charged regions are indicated in blue and negatively charged regions in red. Docking simulations were performed using Surflex-Dock 2.6 program (19, 20).

Potential STAT1-targeting inhibitors were selected from a CL library, using the pre-screen algorithm (see Materials and Methods), according to STAT1-BS. Compounds with the highest STAT1-BS were checked for availability and 12 of them were purchased (Table 2). These compounds, named A01 to L01, displayed STAT1-BS from 8.51 for J01 (the highest) to 7.56 for A01 (the lowest). To test the inhibitory capacity of these compounds toward STAT1 phosphorylation *in vitro*, we first treated HMECs with LPS (1 μg/ml for 4 h) in the presence or absence of the individual compounds (200 μM for 40 h). Except for C01, E01, and F01 (Figures 1A,B), none of the other compounds were able to inhibit STAT1 phosphorylation (not shown). A representative experiment is shown in Figure 1A, in which the phosphorylation and expression of STAT1, was followed. Indeed, a dramatic reduction in phosphorylation, but not total expression, of STAT1 could be observed in LPS-stimulated cells pre-treated with C01, E01, or F01. Notably, treatment with C01 resulted in partial inhibition, whereas E01 and F01 completely inhibited STAT1 phosphorylation (Figure 1B). Under similar conditions, 100 μM of E01 and F01 only partially inhibited STAT1 phosphorylation, while in case of 50 or 25 μM no inhibition could be observed (data not shown). Next, we examined the *in silico* binding affinity of C01, E01, and F01 to the SH2 domain of STAT1, including the pTyr-binding pocket (pY + 0) and the hydrophobic pocket (pY-X). C01 (ZINC08344970, structure shown in Figure S1) exhibited binding affinity to pY+0 and pY-X of STAT1 (Figure 1C), in the same way as F01 (ZINC13362660, structure shown in Figure S1). On the other hand, E01 (ZINC09970661, structure shown in Figure S1) only showed affinity for pY+0, but not to pY-X and shifted toward the Ile-binding sub-site of the STAT1 pTyr-linker (Figure 1C). STAT1-BS was the highest for E01 (8.36) as compared to C01 (8.09) and F01 (7.78). Among these three compounds C01 displayed a higher input of polar interactions to the BS (6.9), than E01 (6.05) and F01 (6.62), but at the same time the highest error rate of binding, represented by Crash value of −1.66. E01 and F01 had significantly lower penalty score for inappropriate binding to STAT1-SH2 domain, −1.27 and −1.04 respectively (Table 2). Together, this suggested that C01, E01, and F01 inhibit STAT1 phosphorylation by targeting the pY+0 and pY-X of its SH2 domain, however with low potency.

**Table 2:** Docking characteristics (pscreen algorithm, STAT1-BS, Crash, and Polar Score) of top 12 selected compounds from Clean Leads primary screen bound to STAT1-SH2 domain.

	**ZINC ID**	**STAT1-BS**	**Crash**	**Polar score**
A01	ZINC04943450	7.56	−1.12	6.96
B01	ZINC05362485	7.71	−1.66	8.67
C01	ZINC08344970	8.09	−1.66	6.9
D01	ZINC09418732	7.63	−1.11	7.57
E01	ZINC09970661	8.36	−1.27	6.05
F01	ZINC13362660	7.78	−1.04	6.62
G01	ZINC13443544	7.81	−1.77	4.94
H01	ZINC15772297	7.79	−0.99	5.98
I01	ZINC20069236	7.76	−1.72	8.99
J01	ZINC20312047	8.51	−0.85	6.87
K01	ZINC07047084	7.58	−1.4	8.94
L01	ZINC08477975	7.66	−1.32	6.92

### C01L_F03 exhibits similarity to C01 and shows potent STAT-SH2 cross-binding

To identify more potent variants of the above characterized STAT1 inhibitors, a similarity screen on the CL list and the CDL list of the ZINC database was performed for compounds with a similarity of 50% to C01, E01, or F01. Moreover, to target multiple sites of the SH2 domain only CDL compounds with a molecular weight >300 g/mol were included. Altogether 1961 compounds were analyzed for C01, E01, and F01 similarity by Surflex-Sim 2.6 and docked to the SH2 domains of STAT1, STAT2, and STAT3, using the more accurate screening method with pgeom parameter settings in Surflex-Dock 2.6 (20). The compounds were filtered by STAT1-CBAV(STAT2) >0 and STAT1-CBAV(STAT3) >0 to compare binding affinities between STAT1, 2, and 3.

**Figure 2:**
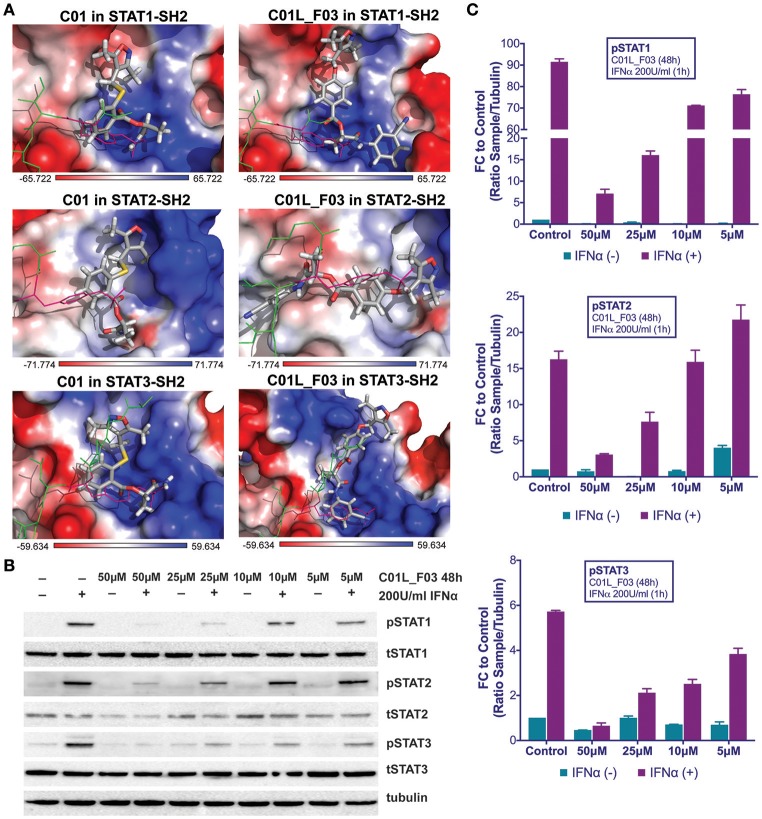
Top-scored binding conformations of C01 and C01L_F03 in the SH2 domain of STAT1, STAT2, and STAT3 **(A)**. C01 and C01L_F03 compounds are shown in stick representation and colored according to the atomic structure. pTyr-linker is represented by green lines with pink pTyr residue. SH2 domains of STAT1, 2 and 3 in the surface representation are colored based on the distribution of the APBS electrostatic surface potential (43). Positively charged regions are indicated in blue and negatively charged regions in red. Docking simulations were performed using Surflex-Dock 2.6 program (19, 20). C01L_F03 inhibits IFNα induced phosphorylation of STAT1, STAT2 and STAT3 **(B)**. HMECs were treated with 50, 25, 10, and 5 μM C01L_F03 for 48 h and with 200 U/ml of IFNα for 1 h. Protein extracts were collected and levels of pSTAT1, pSTAT2, pSTAT3, tSTAT1, tSTAT2, tSTAT3 and α-tubulin were assessed by western blotting. Western quantification **(C)**. Bars represent mean quantification form 3 individual repeats ± SEM as error bars.

Two important observations could be made from the data analysis. First, the most interesting compounds were all from the CDL list with a molecular weight exceeding 300 g/mol. Second, these compounds were mostly C01-like. Accordingly, six compounds C01L_A03 to C01L_F03 (accompanying structures are shown in Figure S2) were selected with STAT1-CBAV >0 (Table 3). Their STAT1-BS as well as their STAT1-CBAV values were higher than those for E01 and F01, calculated using the same pgeom parameter setting. In comparison to C01 all six compounds displayed significant higher STAT1-BS, but in case of STAT1-CBAV differences were not always observed. For example, C01L_A03 contained the highest STAT1-BS (9.84), as well as STAT1-CBAV (3.6 for STAT2 and 4.14 for STAT3). In the docking model of STAT1 C01L_A03 bound to pY+0, pY-X and partially the Ile sub-site (data not shown). C01L_C03, on the other hand, bound to pY+0, while pY-X and the Ile sub-site of STAT1 were only partially targeted (data not shown). In case of C01-similarity, C01L_D03 and C01L_A03 displayed highest C01-SIM values, 0.846 and 0.785, respectively. C01L_E03 and C01L_F03 had more similar structures in comparison to the other compounds (see Figure S2). This correlated with similar STAT1-BS and C01-SIM values for these two compounds (Table 3). Moreover, their binding position in STAT1-SH2 was also comparable (data not shown). A more exhaustive docking analysis was performed for C01L_F03 (ZINC05312694, structure shown in Figure S2). The C01L_F03‘s STAT1-BS of 8.23 and a STAT1-CBAV(STAT3) < 1 (0.22), suggested STAT1 and STAT3-SH2 cross-binding (Table 3). This coincides with the high conservation between these two STATs, sharing 50% of global amino acid sequence homology, according to pairwise sequence identity analysis (44). On the other hand, the higher STAT1-CBAV(STAT2) for C01L_F03 (Table 3; 3.36) predicted lower affinity for STAT2 than for STAT1 and STAT3. In contrast, the C01 compound displayed similar STAT1-CBAV for STAT2 and STAT3, 1.74 and 2.08 respectively, whereas the STAT1-BS was lower by 1.5 than for C01L_F03 (Table 3). The binding affinity of C01L_F03 to the individual STAT-SH2 domains, corresponded with the graphical analysis. According to Table 3, from the top 20 optimized binding conformations of C01L_F03 to STAT1-SH2, 19 (95%) favored pY+0 and pY-X simultaneously. LBPV analyses for other STAT-SH2 revealed that C01L_F03 also shares high affinity for pY+0 and pY-X in case of STAT3 with LBPV_0+X_ = 0.75 and much lower for STAT2 with LBPV_0+X_ = 0.2 (Table 4). These calculations were supported by graphical presentation of the docking results (Figure 2A) in which the top scored conformation of C01L_F03 for each individual STAT competed with pTyr binding to the particular STAT-SH2 domain. In the docking model of STAT1-SH2, C01L_F03 bound to pY+0 and pY-X similar to C01. The same conformation could be observed in the STAT3-SH2. In case of STAT2-SH2, C01L_F03 predominantly bound to pY+0, but not to pY-X and shifted toward the Leu-binding sub-site of the STAT2 pTyr-linker (Figure 2A). C01 in the STAT2-SH2 domain, on the other hand, remained in a similar position as in STAT1 and STAT3-SH2 domains. Together, the docking results of C01 and C01L_F03 suggest higher potency of the latter toward STAT1 inhibition, although with a certain degree of STAT-SH2 cross-binding.

**Table 3:** Docking characteristics (pgeom algorithm, STAT1-BS, STAT1-CBAV) of C01L_A03-C01L_F03 bound to STAT1, STAT2, and STAT3-SH2 domain in comparison to C01, E01 and F01 from primary screening, as well as C01-like similarity analysis (C01-SIM, C01-RMSD).

**Compound**	**ZINC ID**	**C01-SIM**	**C01-RMSD**	**STAT1-BS**	**STAT1-CBAV (STAT2)**	**STAT1-CBAV (STAT3)**
C01	ZINC08344970	1.000	0.00	6.73	1.74	2.08
E01	ZINC09970661	0.604	2.96	6.95	−1.65	−0.73
F01	ZINC13362660	0.567	2.40	6.38	−0.09	−0.19
C01L_A03	ZINC03470000	0.785	2.28	9.84	3.60	4.14
C01L_B03	ZINC05585448	0.771	2.82	8.31	2.19	1.19
C01L_C03	ZINC08712870	0.760	2.47	7.08	0.70	1.00
C01L_D03	ZINC08712921	0.846	2.84	7.30	1.50	1.78
C01L_E03	ZINC21128441	0.777	2.13	8.01	2.94	2.68
C01L_F03	ZINC05312694	0.767	2.75	8.23	3.36	0.22

**Table 4:** Docking characteristics (pgeom algorithm, STAT1-BS, STAT1-CBAV, LBPV) of STATTIC, STX-0119, C01, E01, F01, and C01L_F03 bound to STAT1, STAT2, and STAT3-SH2 domain.

**Compound**	**STAT1-BS**	**STAT1-CBAV (STAT2)**	**STAT1-CBAV (STAT3)**	**STAT1-LBPV**		**STAT2-LBPV**		**STAT3-LBPV**	
STATTIC	4.70	−0.86	0.39	0.7_0_	0.2_X_	0.9_0_	0.05_X_	0.3_0_	0.55_X_
STX-0119	4.36	−0.32	0.25	0.45_0+X_		0.3_0+X_		0.25_0+X_	
C01	6.73	1.74	2.08	0.5_0+X_		0.45_0+X_		0.35_0+X_	
E01	6.95	−1.65	−0.73	0.65_0+X_		0.3_0+X_		0.3_0+X_	
F01	6.38	−0.09	−0.19	0.2_0+X_		0.2_0+X_		0.3_0+X_	
C01L_F03	8.23	3.36	0.22	0.95_0+X_		0.2_0+X_		0.75_0+X_	

### C01L_F03 inhibits IFNα and IL-6 induced phosphorylation of STAT1, STAT2 and STAT3 and binding to target gene promoters and their expression

In addition to the docking experiments, the six compounds from the similarity screen were tested for their potential to block IFNα induced STAT phosphorylation. This led to the selection of C01L_F03 as our most potent candidate (not shown).

**Figure 3:**
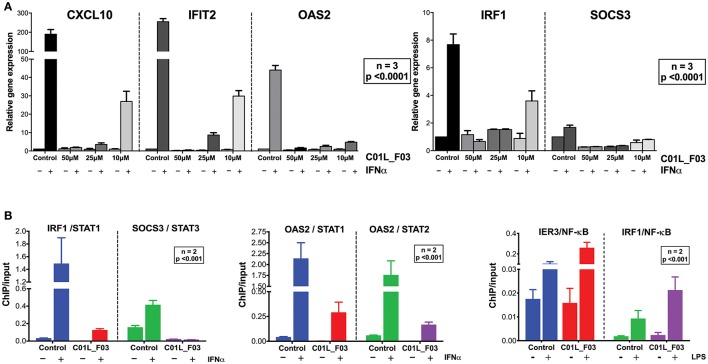
C01L_F03 inhibits IFNα induced gene expression of CXCL10, IFIT2, OAS2, IRF1, and SOCS3 **(A)**. HMECs were treated with 50, 25, 10 of C01L_F03 for 48 h and with 200 U/ml of IFNα for 4 h. RNA was isolated and subjected to qPCR analysis. Experiments were performed in 3 individual repeats, which were compared by two-way ANOVA test and unpaired two-tailed student *T*-test. C01L_F03 inhibits IFNα stimulated binding of STAT1, STAT2 and STAT3 to the ISRE of OAS2, and GAS of IRF1 and SOCS3, and LPS stimulated binding of p65 to the NF-κB binding site of IER3 and IRF1 **(B)**. HMECs were treated with 50 μM of C01L_F03 for 48 h. Chromatin was isolated and subjected to IP with antibodies against pSTAT1, pSTAT2, pSTAT3 or NF-κB (p65), followed by qPCR analysis (primers are listed in Table 1). Experiments were performed in 2 individual repeats, which were compared by two-way ANOVA test and unpaired two-tailed student *T*-test.

To address STAT cross-binding specificity of C01L_F03 we pre-treated HMECs for 48 or 24 h (data not shown) with various concentrations of the compound (50, 25, and 10 μM) in the presence or absence of IFNα (200 U/ml) or IL-6 (100 ng/ml), which were added 1 h before protein isolation. A representative experiment is shown in Figure 2B and Figure S3 in which the phosphorylation of STAT1, STAT2, and STAT3 was followed. These results stand in line with our docking studies. IFNα induced phosphorylation of all three STATs was almost fully inhibited in the presence of 50 μM and 25 μM, and partially of 10 μM and 5 μM of C01L_F03 for 48 h (Figure 2C). A similar inhibition pattern was observed for STAT3 phosphorylation upon IL-6 treatment (Figure S3). Twenty-Four hour treatment with C01L_F03 resulted only in partial inhibition with the highest concentration (data not shown). Under the same conditions levels of total STAT proteins were not influenced by C01L_F03 treatment. After 48 h treatment with 50 μM C01L_F03 exhibited cytotoxic effect causing death of approximately 30% of cells (not shown). However, at a concentration of 25 μM barely any toxicity was visible. What is more 24 h treatment with 50 μM of C01L_F03 showed no toxicity (not shown). The inhibitory effect of C01L_F03 was also studied at the gene expression level. In agreement with the STAT cross-binding characteristics from our docking results, C01L_F03 was able to completely inhibit IFNα-induced expression of the multiple STAT- and IRF-target genes, CXCL10, IFIT2, and OAS2 at 25 μM and partially for 10 μM pre-treated for 48 h (Figure 3A). The same was true for the STAT1 target gene, IRF1 and the STAT3 target gene, SOCS3. To further demonstrate that the effect of C01L_F03 on STAT target gene expression was mediated by inhibiting binding of STATs to target gene promoters, we performed immunoprecipitation followed by qPCR on Chromatin extracted from untreated or IFNα treated HMECs in the absence or presence of 50 μM C01L_F03. Accordingly, using antibodies against pSTAT1, pSTAT2, or pSTAT3, treatment with IFNα caused enhanced binding of pSTAT1 and pSTAT2 to the promoter ISRE element of OAS2 (Figure 3B), and respectively of pSTAT1 and pSTAT3 to IRF1 and SOCS3 containing GAS sites as compared to untreated controls (Figure 3B). More important, the presence of CO1L_F03 dramatically reduced this DNA-binding of the different STATs (Figure 3B) and correlated with inhibition of target gene expression (Figure 3A).

### Stattic and STX-0119 exhibit STAT cross-binding in analogy to C01L_F03

**Figure 4:**
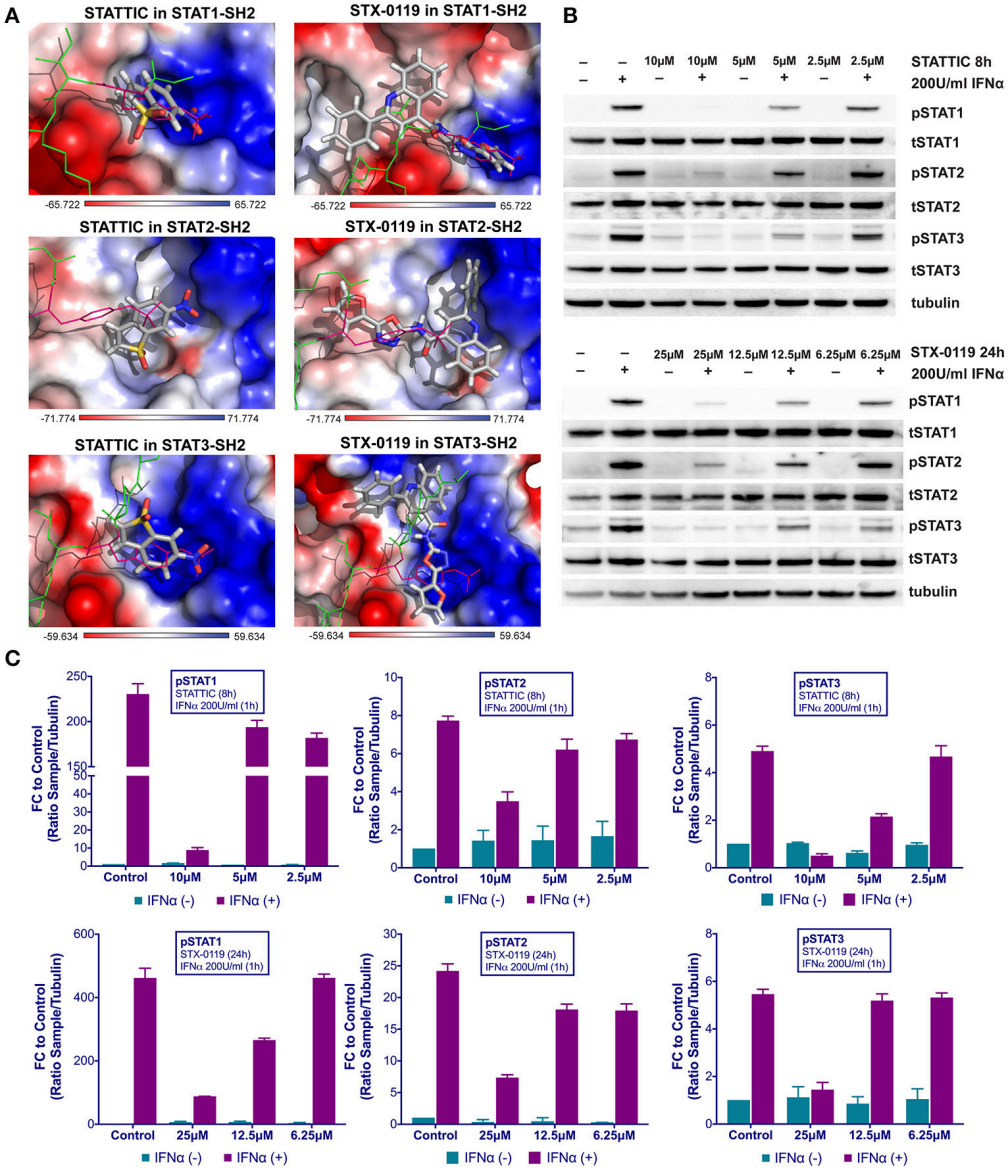
Top-scored binding conformations of STATTIC and STX-0119 in the SH2 domain of STAT1, STAT2, and STAT3 **(A)**. STATTIC and STX-0119 compounds are in stick representation and colored according to the atomic structure. pTyr-linker is represented by green lines with pink pTyr residue. SH2 domains of STAT1, 2 and 3 in the surface representation are colored based on the distribution of the APBS electrostatic surface potential (43). Positively charged regions are indicated in blue and negatively charged regions in red. Docking simulations were performed using Surflex-Dock 2.6 program (19, 20). STATTIC and STX-0119 inhibit IFNα induced phosphorylation of STAT1, STAT2 and STAT3 **(B)**. HMECs were treated with 10, 5, 2.5 μM of STATTIC for 8 h or with 25, 12.5, 6.25 μM of STX-0119 for 24 h and with 200 U/ml of IFNα for 1 h. Protein extracts were collected and levels of pSTAT1, pSTAT2, pSTAT3, tSTAT1, tSTAT2, tSTAT3 and α-tubulin were assessed by western blotting. Western quantification **(C)**. Bars represent mean quantification form 3 individual repeats ± SEM as error bars.

Recently, we proposed a similar STAT cross-binding mechanism for STATTIC and STX-0119 (16), chemical structures of STATTIC and STX-0119 are displayed in Figure S1. They were previously discovered as direct STAT3 inhibitors by high throughput screening (13) and virtual screening (25), respectively. In analogy to C01L_F03, we decided to examine this in more detail, by using a comparative docking strategy combined with western and Real-time PCR analysis. Using the pgeom algorithm, docking simulation of STATTIC and STX-0119 in the STAT1, 2 and 3 SH2 domain resulted in 20 optimized conformations for each compound. Moreover, corresponding BS values were calculated for each individual STAT (not shown). Table 3 shows the top STAT1-BS of STATTIC (4.70) and of STX-0119 (4.36), as well as STAT1-CBAVs (STATTIC: −0.86 for STAT2 and 0.39 for STAT3; STX-0119: −0.32 for STAT2 and 0.25 for STAT3). As becomes clear from the calculated STAT1-CBAVs, both compounds exhibited nearly identical binding affinity to the STAT1, 2 and 3 SH2 domain. In addition, STATTIC and STX-0119 LBPV toward the STAT-SH2 pY+0 and pY-X cavities were determined. Thus, the conformational tendencies of STATTIC and STX-0119 to the STAT3-SH2 were calculated. According to Table 4, from the top 20 optimized binding conformations of STATTIC to STAT3-SH2, 6 (30%) favor pY+0 and 11 (55%) fit to pY-X. LBPV analyses for other STAT-SH2 revealed that STATTIC also shares partial affinity between pY+0 and pY-X in case of STAT1 and STAT2 (Table 4) similar to STAT3. From the top 20 optimized binding conformations of STX-0119 to STAT3-SH2, only 5 (25%) of them favor both cavities simultaneously, which is in the same range in case of STAT1 and STAT2 (Table 4). Graphical analysis of the docking results (Figure 4A) was performed as a supplement to the numerical values. For each individual STAT the top scored conformation of STATTIC and STX-0119 competes with pTyr in binding to the STAT-SH2 domain. Due to its small size and low molecular weight STATTIC lacks STAT-SH2 binding specificity, which is supported by our recent docking results. Because of targeting both cavities (pY-0 and pY-X) with low BS difference (based on CBAV) and weak affinity (based on LBPV) the same is true for STX-0119.

### Stattic and STX-0119 inhibit IFNα-induced STAT1, STAT2 and STAT3 phosphorylation and target gene expression

**Figure 5:**
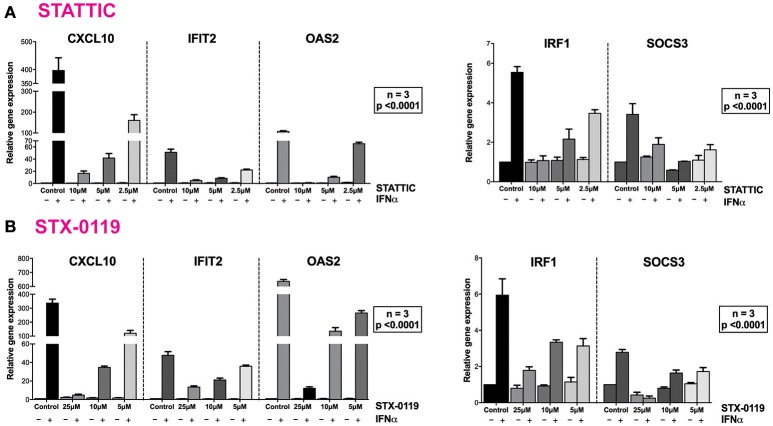
STATTIC and STX-0119 inhibits IFNα induced gene expression of CXCL10, IFIT2, OAS2, IRF1, and SOCS3. HMECs were treated with **(A)** 10, 5, 2.5 μM of STATTIC for 8 h or **(B)** with 25, 12.5, 6.25 μM of STX-0119 for 24 h and with 200 U/ml of IFNα for 4 h. RNA was isolated and subjected to qPCR analysis. Experiments were performed in 3 individual repeats, which were compared by two-way ANOVA test and unpaired two-tailed student *T*-test.

These findings were further validated in HMECs *in vitro*, by testing the potential of STATTIC and STX-0119 at varying concentrations to inhibit STAT phosphorylation induced by IFNα. For STATTIC and STX-0119 (Figures 4B,C), we observed inhibition of phosphorylation of STAT1, STAT2 and STAT3 in a concentration dependent manner (STATTIC: between 10 and 2.5 μM for 8 h; STX-0119: between 25 and 6.25 μM for 24 h). Corresponding with the effects shown at the STAT-phosphorylation level both inhibitors also efficiently decreased IFNα-induced gene expression of the multi-STAT and IRF-targets CXCL10, OAS2 and IFIT2, the STAT1-only target IRF1 and STAT3-only target SOCS3 (Figures 5A,B). In comparison to C01L_F03, STATTIC was the most potent one of the three tested compounds. Moreover, all three compounds exhibited a certain degree of cytotoxicity only at the highest used concentrations (not shown).

Together our data provide a molecular basis for STAT-cross-binding specificity of C01L_F03, STATTIC, and STX-0119 and their potential to inhibit multi-STAT and IRF-target genes.

### C01L_F03, stattic and STX-0119 interact with the SH2 domain of STAT1, STAT2, and STAT3

**Figure 6:**
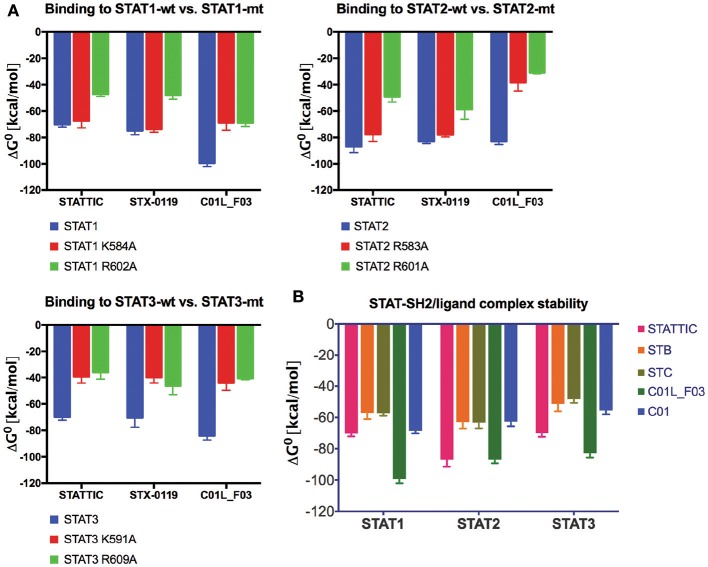
Comparison of wt and mutated STAT-SH2/ligand complex stability **(A)**. STAT1, 2 or 3, wild type and with single point mutation to Ala, complexes with STATTIC, STX-0119 or C01L_F03 have been subjected to the *in silico* studies of binding stability in the equilibrium. ΔG^0^ (free enthalpy change), which is a measure of the strength of the complex formation was estimated [based on HADDOCK ligand docking protocol (26, 27) with addition of Surflex-Dock protocol (16)]. More negative ΔG^0^ (higher free enthalpy change) corresponds to stronger interaction between ligand and the protein, which reflects better complex stability. Comparison of STAT-SH2/ligand complex stability **(B)**. Complexes of STAT1, 2 and 3, with STATTIC and its analogs STB and STC, as well as C01 and C01L_F03 have been subjected to the *in silico* studies of binding stability in the equilibrium as in **(A)**.

To provide evidence that STATTIC, STX-0119 and C01L_F03 concert their inhibitory actions through direct interaction with the STAT-SH2 domain we performed docking simulations in combination with STAT-SH2 *in silico* mutagenesis (see Materials and Methods). As presented in Figure 6A, mutating a.a. R602 in STAT1 (45), R601 in STAT2 (46) and R609 in STAT3 (47), resulted in a significant decrease in binding stability (ΔG^0^) between the SH2 domains of STAT1, STAT2 and STAT3 and all three inhibitors. The same was true, after mutating a second important a.a. K584 in STAT1 (48), R583 in STAT2 and K591 in STAT3 (18), albeit to a lesser extent (Figure 6A). A similar approach was used to compare binding stability of STATTIC and published STATTIC analogs, STB and STC, Figure S1 (13), and of C01L_F03 and C01 which differ in *in silico* binding affinity for STAT1, 2, and 3 (Table 4). As becomes clear from Figure 6B, STATTIC analogs exhibit lower binding stability (ΔG^0^) for the SH2 domains of STAT1, 2 and 3 as compared to wt STATTIC. Likewise, interaction between C01 and STAT1, 2 and 3, corresponds with a lower binding stability (ΔG^0^) in relation to C01L_F03.

### C01L_F03, STATTIC and STX-0119 commonly inhibit cross-talk between IFNγ and LPS in a “multi-STAT” and “STAT-only” manner

**Figure 7:**
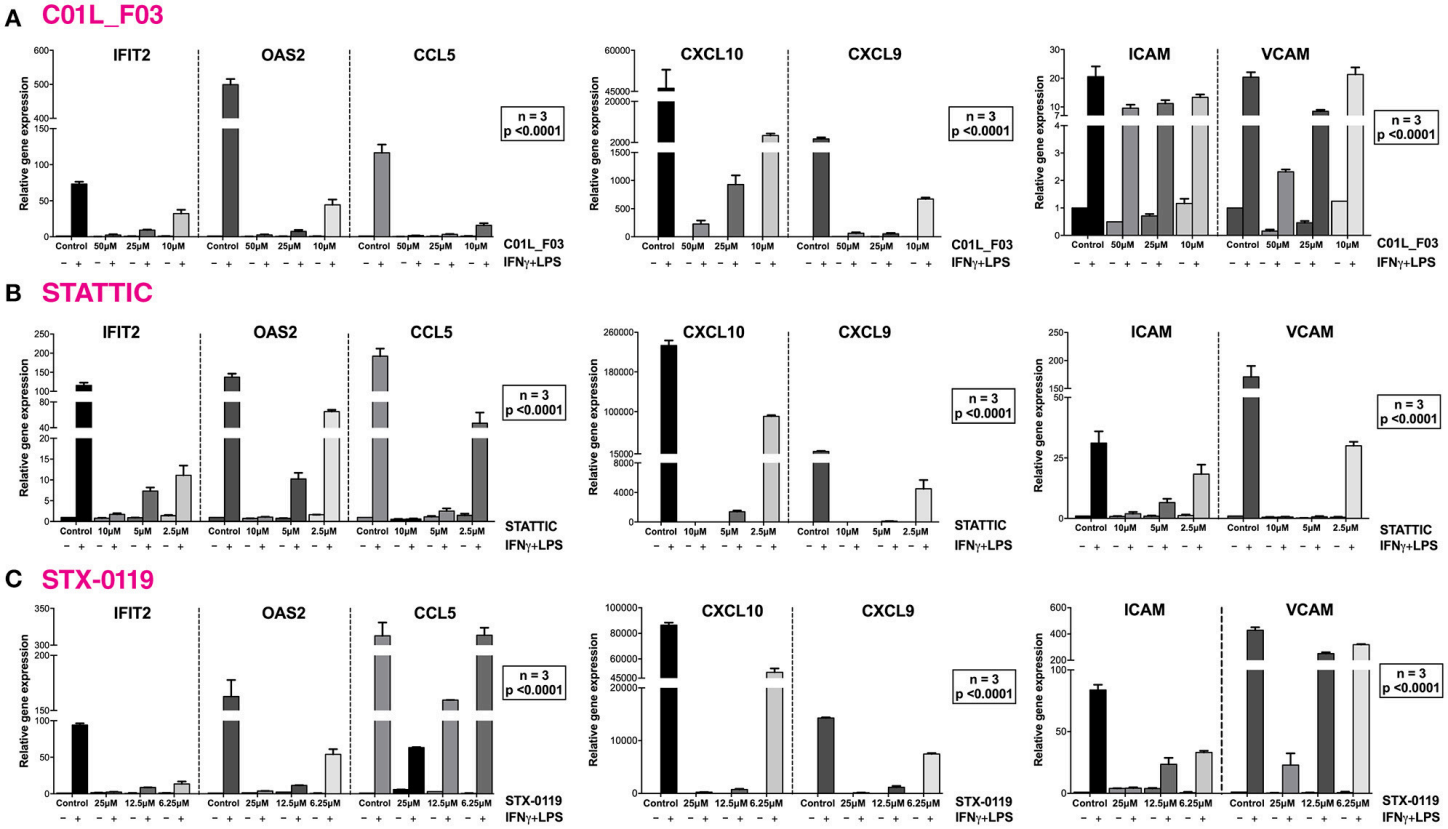
C01L_F03, STATTIC and STX-0119 inhibit IFNγ+LPS induced gene expression of IFIT2, OAS2, CCL5, CXCL10, CXCL9, ICAM1, and VCAM1. HMECs were treated with **(A)** 50, 25, 10 μM of C01L_F03 for 48 h or **(B)** 10, 5, 2.5 μM of STATTIC for 8 h or **(C)** with 25, 12.5, 6.25 μM of STX-0119 for 24 h and for 8 h with IFNγ + 4 h with LPS. RNA was isolated and subjected to qPCR analysis. Experiments were performed in 3 individual repeats, which were compared by two-way ANOVA test and unpaired two-tailed student *T*-test.

In a second set of experiments HMECs were treated with IFNγ and LPS to further investigate the ability of C01L_F03, STATTIC, and STX-0119 to inhibit pro-inflammatory and pro-atherogenic signaling depending on multiple STATs, IRFs and NF-κB. As shown in Figure 7, pre-treatment of HMECs with C01L_F03, STATTIC or STX-0119 resulted in inhibition of IFNγ+LPS induced gene expression of IFIT2, OAS2, CCL5, CXCL10, CXCL9, ICAM1 and VCAM1, in a concentration dependent manner. Pre-treating HMECs with IFNγ+LPS, followed by STATTIC, or simultaneous treatment of IFNγ+LPS with C01L_F03 or STX-0119 (representing a more therapeutic mode), likewise resulted in potent inhibition of CXCL10, IFIT2, and OAS2 expression (Figure S4). In general, the different compounds displayed similar inhibition characteristics, although sometimes minor variations could be observed. These data suggested that C01L_F03, STATTIC and STX-0119 may commonly block STAT cooperative promotor activation with IRF and NF-κB mediated by IFNγ and LPS in human microvascular endothelial cells. To provide further evidence for this, we decided to study the genome-wide effect of C01L_F03, STATTIC and STX-0119 on IFNγ+LPS-mediated vascular inflammation. For this, we performed a microarray experiment on RNA isolated from HMECs treated with IFNγ+LPS in the presence or absence of: 50 μM of C01L_F03, 25 μM of STX-0119 or 10 μM of STATTIC (GEO accession: GSE101508). IFNγ+LPS increased the expression of 731 genes at least two-fold or higher as compared to untreated cells, of which the top-25 are shown in Table 5.

**Table 5:** Representative top-25 genes induced by IFNγ+LPS, displaying significant inhibition by all three compounds.

**Gene id**	**Fold change relative to control**			
	**IFNγ+LPS**	**C01L_F03**	**STATTIC**	**STX-0119**
CXCL10	9298.61	615.79	15.14	223.90
CCL8	2118.86	6.83	6.31	0.79
UBD	1860.83	129.28	2.09	62.19
CXCL9	1565.00	2.26	1.00	6.31
GBP4	793.16	38.79	16.30	8.61
GBP5	435.89	20.26	5.26	11.63
OAS2	329.65	2.61	6.29	1.48
VCAM1	326.83	33.01	1.36	8.12
CCL7	289.01	1.00	3.41	1.00
INDO	209.33	1.00	1.80	1.00
MMP3	192.62	2.22	28.96	2.89
IDO1	178.80	1.62	6.03	1.42
CCL3L3	165.18	4.69	1.52	20.25
OASL	160.22	3.99	1.21	14.16
LYPD5	127.07	6.75	6.90	7.66
LTB	125.33	1.00	0.75	0.95
MMP12	111.79	0.70	0.70	0.70
IFI44L	100.04	1.14	1.14	1.21
LOC730249	91.23	0.60	0.96	0.78
CD74	88.67	1.18	0.76	0.84
LOC100129681	76.64	1.31	2.35	0.18
MX2	68.99	0.40	0.74	1.23
DLL1	68.78	3.39	10.80	8.11
C1S	64.57	4.22	5.98	2.39
TNFSF10	64.45	0.67	1.22	0.21

*Gene expression levels were presented as fold change relative to control*.

**Figure 8:**
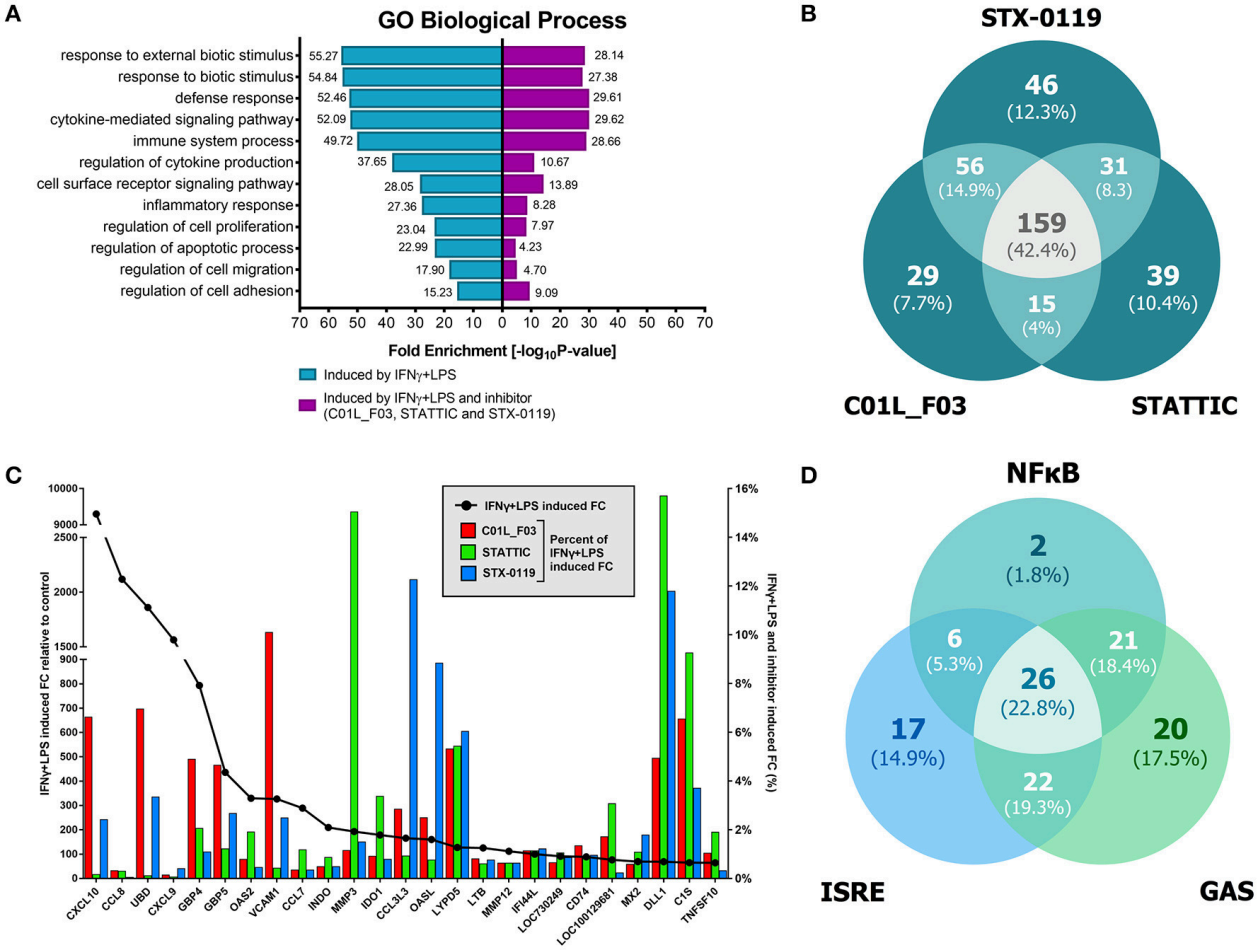
Comparison of top GO terms **(A)**. Terms were selected based on Fold Enrichment values, between genes induced by treatment with IFNγ+LPS and group of IFNγ+LPS induced and inhibited simultaneously by three compounds. Venn diagram distribution of IFNγ+LPS induced and inhibited (C01L_F03, STATTIC, STX-0119) genes **(B)**. Data were obtained from HMECs treated with 50 μM of C01L_F03, 10 μM of STATTIC and 25 μM of STX-0119 and IFNγ+LPS. Three lists of inhibited genes were uploaded and analyzed by VennDiagram package in R (31). The diagram shows how many genes are induced by IFNγ+LPS and simultaneously inhibited by two or three inhibitors or only by one compound. Representative genes induced by IFNγ+LPS, displaying significant inhibition by all three compounds **(C)**. Expression levels of IFNγ+LPS-induced genes (displayed as •, left Y-axis) were presented as fold change (FC) relative to control. Expression of C01L_F03 or STATTIC or STX-0119 inhibited genes (displayed as colored bars, right Y-axis) was presented as percent of IFNγ+LPS-induced FC. Venn diagram distribution of ISRE, GAS, NF-κB binding sites among genes inhibited simultaneously by three compounds **(D)**. Three lists of inhibited genes were uploaded and analyzed by VennDiagram package in R (31).

These included many known IFNγ and LPS target genes associated with: chemotaxis/migration (CXCL9, CXCL10, CCL7, CCL8, CCL3L3, MMP3, MMP12), adhesion (VCAM1, CD74), immune response to viral infection (UBD, GBP4, GBP5, OAS2, MX2, INDO, OASL, IFI44L, MX2). GO analysis of the complete list of IFNγ+LPS responsive genes revealed enrichment of biological functions mainly involved in: cytokine-mediated signaling pathway (GO:0019221); defense response and immune system process (GO:0006952 and GO:0002376); regulation of cytokine production (GO:0001817), inflammatory response (GO:0006954), regulation of cell adhesion (GO:0030155) or cell migration (GO:0030334), (see Table 5, Figure 8A).

Next, we aimed at identifying the IFNγ+LPS target genes that were commonly inhibited by C01L_F03, STX-0119 and STATTIC. For this, genes were considered of which the expression was more than 4 times inhibited by all three inhibitors as compared to IFNγ+LPS alone (see Materials and Methods). As such, out of the 731 up-regulated genes C01L_F03 inhibited expression of 259, STATTIC of 244 genes and STX-0119 of 292 genes (Figure 8B). What is more, expression of 159 genes was commonly inhibited by C01L_F03, STATTIC and STX-0119 (according to the requirements specified in Materials & Methods), of which the inhibition pattern of the top-25 is displayed in Figure 8C. Among those we could recognize the ones which were already validated by Real-time PCR (Figures 3A, 5A,B, 7) e.g., CXCL10, IFIT2, OAS2, or VCAM1, as well as many known STAT target genes (i.e., SOCS1, IRF1, IRF8, APOL1, BID as STAT1 targets; IFIT1, IFIT2, IFIT3, OAS1, OAS2, MX1, MX2, ISG15 as STAT1-STAT2 targets; SOCS3, CCND1, MMP3, FAS PIM1, VEGF, S1PR1 as STAT3 targets). GO analysis of the 159 commonly inhibited genes furthermore revealed enrichment of biological functions connected to pro-inflammatory and pro-atherogenic responses (Figure 8C). The complete list of up and down-regulated genes in response to IFNγ+LPS in the presence or absence of C01L_F03, STATTIC and STX-0119 is shown in Table S1.

To address the “multi-STAT” and “STAT-only” characteristics of these three inhibitors, we subsequently performed promoter analysis on the 159 commonly inhibited genes, for the presence of ISRE, STAT or NF-κB binding sites. Figure 8D shows the predicted representation of individual or combined ISRE, STAT or NF-κB binding sites, in their proximal promoters (−950 to +100). The majority of these genes contained single ISRE (14.9%) or GAS (17.5%) sites, or combinations of ISRE+GAS (19.3%), ISRE+NF-κB (5.3%), GAS+NF-κB (18.4%) or ISRE+GAS+NF-κB (22.8%). In general, under these conditions ISRE motifs correspond to potential binding of multiple STATs (STAT1 and STAT2) and IRFs (IRF1, IRF8, and IRF9), and GAS motifs to that of multiple STATs (STAT1 and STAT3). Surprisingly, 2 genes (1.8%), IL7R, USP18 were assigned to the group with only an NF-κB site in their proximal promoter. However, both genes contained either a GAS (IL7R) or ISRE (USP18) sequence just outside the 950 bp selected promoter area (not shown). To further proof that DNA binding of NF-κB (p65) was not affected under these conditions we performed ChIP qPCR on genes containing either both STAT1 and NF-κB binding sites (IRF1) or only an NF-κB binding element (IER3) (Figure 3B). Indeed, C01L_F03 did not effect the LPS-induced DNA binding of p65 to the promoter of these two genes, which correlated with the partial (IRF1) or lack of inhibition (IER3) of their expression as observed in our microarray experiment (Table S1). These results strongly suggest that C01L_F03, STATTIC and STX-0119 are “multi-STAT” and “STAT-only” inhibitors that commonly inhibit pro-inflammatory and pro-atherogenic gene expression directed by cooperative involvement of STATs with IRFs and/or NF-κB.

### C01L_F03, STATTIC and STX-0119 inhibit IFNγ+LPS induced VSMC migration

**Figure 9:**
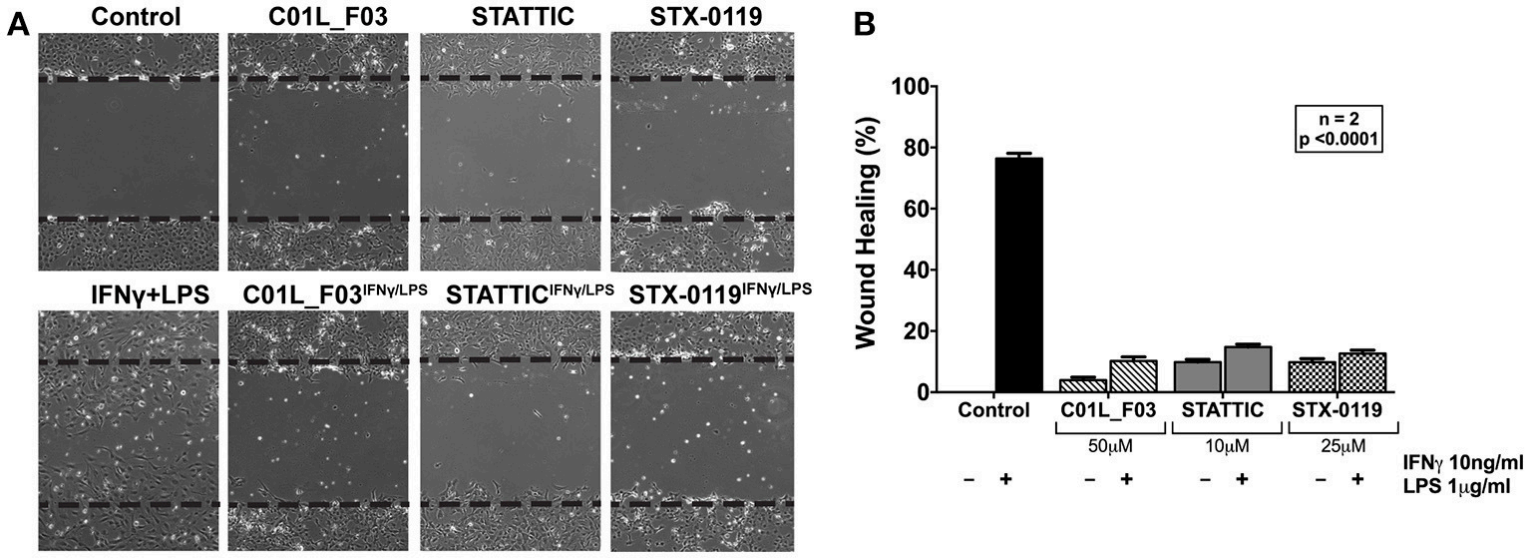
Wound healing assay performed on HMECs treated with C01L_F03, STATTIC and STX-0119 with or without IFNγ+LPS presence **(A)**. Dashed lines determine scratch borders at the beginning of the experiment. Statistical evaluation of wound healing assay **(B)**. Graph shows percentage of healed wound in comparison to 0 h control. Experiment was performed in 2 individual repeats (40 distance measurements in total), which were compared by two-way ANOVA test.

In addition, we aimed at providing evidence that a multi-STAT inhibitory strategy could be used to inhibit IFNγ+LPS induced vascular inflammation in different models. First, we performed a wound healing assay to examine the effect of all three compounds on IFNγ+LPS induced ECs migration (Figure 9A). Cells stimulated with IFNγ+LPS showed increased capacity of migration, resulting in almost 80% wound coverage after 12-h of treatment (Figure 9B). In contrast, HMECs treated additionally with C01L_F03, STATTIC or STX-0119 demonstrated drastic reduction of movement. All three inhibitors caused decrease of IFNγ+LPS induced wound healing to less than 15% (Figure 9B), whereas in the absence of IFNγ+LPS they were not capable of closing more than 10% of the artificial wound (Figure 9B). In agreement with the effect on IFNγ+LPS-induced gene expression (Figure 8), based on concentration and time of treatment, STATTIC was the most potent of the three compounds.

### C01L_F03, STATTIC and STX-0119 inhibit IFNγ and LPS induced mononuclear leukocyte adhesion to HUVECs

**Figure 10:**
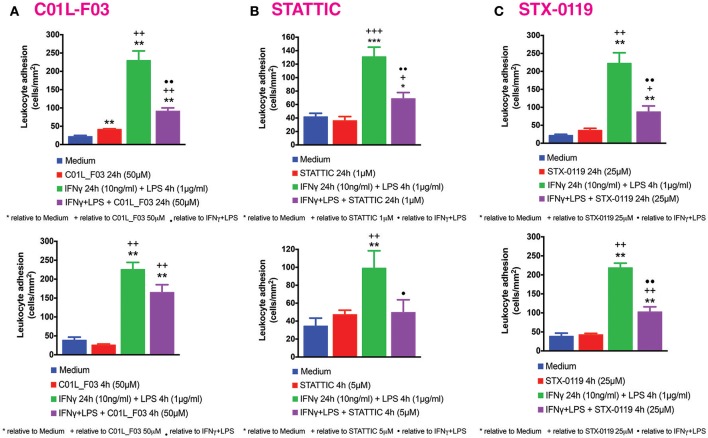
C01L_F03, STATTIC, STX-0119 and inhibit IFNγ+LPS-induced HUVECs mononuclear cell adhesion under physiological flow. HUVECs were stimulated with IFNγ (10 ng/ml) for 24 h and LPS (1 μg/ml) for 4 h. In the experiments, cells were pretreated with **(A)** C01L_F03 50μM for 4 h or C01L_F03 50 μM for 24 h; **(B)** STATTIC 5 μM for 4 h or STATTIC 1 μM for 24 h; **(C)** STX-0119 25 μM for 4 h or STX-0119 25 μM for 24 h. Freshly isolated human mononuclear cells (106 cells/ml) were perfused across the endothelial monolayers for 5 min. at 0.5 dyn/cm^2^ and leukocyte adhesion quantified. Experiments were performed in 5–7 individual repeats, which were compared by one-way ANOVA test and unpaired two-tailed student *T*-test with *p < 0.05; **p < 0.01 and ***p < 0.001.

Our previous studies reported increased adhesion of monocytes to ECs *in vitro* under static conditions in response to IFNγ and LPS in a STAT1-dependent manner (4). We next evaluated the effect of IFNγ for 24 h followed by LPS for another 4 h challenge on mononuclear-endothelial cell interactions *in vitro* using the dynamic flow chamber assay. Thus, freshly-isolated human mononuclear cells were perfused across HUVECs monolayers stimulated or not with the IFNγ+LPS combination. Significant increases in mononuclear cell adhesion were detected in stimulated cells compared to untreated cells (Figure 10). Treatment with C01L_F03 (50 μM; Figure 10A), STATTIC (5 μM; Figure 10B) or STX-0119 (25 μM; Figure 10C) for 4 h or 24 h resulted in significant inhibition of mononuclear cell adhesion to ECs induced by the IFNγ+LPS combination. In the presence of a lower concentration of STATTIC, 1 μM (24 h), a similar drastic reduction in the number of adhered mononuclear cells induced by the IFNγ+LPS combination was observed >70% inhibition, Figure 10B).

### C01L_F03, stattic and STX-0119 protect against IFNγ and LPS induced impairment of mesenteric artery contractility

**Figure 11:**
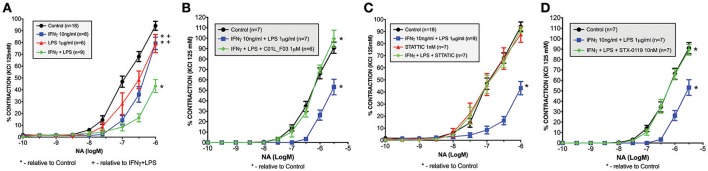
Ameliorated response to noradrenaline in mesenteric arteries stimulated with IFNγ and LPS **(A)**. Isolated mesenteric arteries from WT mice were incubated with IFNγ (10 ng/ml for 3 h prior to NA stimulation), and/or LPS (1 μg/ml for 1.5 h prior to NA stimulation). Next, response to noradrenaline was tested on the small-vessel myograph. C01L_F03, STATTIC and STX-0119 prevent the impaired response to NA elicited by IFNγ+LPS treatment. Isolated mesenteric arteries from WT mice were pre-incubated with **(B)** C01L_F03 (1 μM for 4 h) or **(C)** STATTIC (1 nM for 4 h) or **(D)** STX-0119 (10 nM for 4 h) prior to NA stimulation and/or IFNγ (10 ng/ml for 3 h prior to NA stimulation) and LPS (1 μg/ml for 1.5 h prior to NA stimulation). Next, response to noradrenaline was tested on the small-vessel myograph. Response to noradrenaline in WT mice presented as a percentage of the maximal contraction to KCl. Two-way ANOVA test was used with *p < 0.05 vs. Control and +*p* < 0.05 vs. IFNγ+LPS.

Recently we also observed that among the genes that were highly amplified upon treatment with IFNγ and LPS in primary mouse VSMCs, appeared inducible nitric oxide synthase (iNOS, Nos2). Dysregulation of Nos2 expression and its activity affect vessel function. Thus, we evaluated the physiological ramifications of these experimental conditions using a wire myograph/organ chamber setting. Here we examined the possible protective effect of a multi-STAT inhibitory strategy under similar conditions. As expected, stimulation of the mesenteric arteries isolated from WT animals with IFNγ+LPS resulted in drastic impairment of contractility after subjection to NA treatment as compared to matched control arteries (Figure 11A). Nevertheless, pre-incubation with C01L_F03 (1 μM), STATTIC (1 nM) or STX-0119 (10 nM) prevented the impaired response to NA elicited by IFNγ+LPS (Figures 11B–D respectively). Notably, STATTIC and STX-0119 could only be used in the nM range, without causing IFNγ+LPS-independent impairment of vessel function and integrity (not shown).

## Discussion

Abnormal activation of STAT pathways is present in many human diseases, including CVDs. This fact marks these proteins as highly interesting therapeutic targets (49, 50). By exploring the pTyr-SH2 interaction area of STAT3, searches for STAT3-targeting compounds yielded many small molecules including STATTIC and STX-0119. Only a few inhibitors for other STATs are described. In our pursuit for novel STAT inhibitors, we used a comparative *in silico* docking strategy on CL library from ZINC in combination with 3D structure models for human (h)STAT1, 2 and 3. We selected three novel STAT1 inhibitors C01, E01, and F01 that inhibit STAT1 phosphorylation by targeting the pY+0 and pY-X of its SH2 domain, however with low potency (Figure 1). To find more potent variants of these compounds, a similarity screen on the CL and the CDL libraries of the ZINC database was performed for compounds with a similarity of ≥ 50% to C01, E01 or F01 (Tables 3, 4). Consequently, we identified the novel multi-STAT inhibitor C01L_F03, which targets the SH2 domain of STAT1, STAT2, and STAT3 with the same affinity (Figure 2). In addition, it was shown to simultaneously block phosphorylation and DNA-binding of these three STATs and expression of a selection of target genes in ECs in response to IFNα (Figures 2, 3). These included the multiple STAT (STAT1/STAT2)-target genes, CXCL10, IFIT2 and OAS2, as well as the STAT1 target gene, IRF1 and the STAT3 target gene, SOCS3. It also predicts anti-inflammatory potential of C01L_F03 by simultaneous inhibiting STAT1, STAT2, and STAT3 activity.

According to a similar docking strategy, recently we obtained further insight into the STAT-SH2 cross-binding specificity of a pre-selection of known STAT3 inhibitors, including STATTIC and STX-0119 (16) (Table 4). All the studied compounds targeted the highly conserved pTyr-SH2 binding pocket of all STATs. We concluded, based on the binding affinity scores (BS) and graphic representation in the SH2 domain of hSTAT1, hSTAT2 and hSTAT3, that none of these compounds are STAT3-specific. Here, we followed up on the proposed STAT cross-binding specificity of STATTIC and STX-0119. As compared to C01L_F03, a similar *in silico* and *in vitro* multi-STAT inhibiting capacity was shown for STATTIC and STX-0119 (Figures 4, 5). STATTIC was the most potent of the three compounds, reflected by the time of treatment and concentration used. This could agree with the fact that STATTIC is the smallest compound of the three, and equally targeted the pTyr-binding or hydrophobic SH2 cavity. In addition, the covalent binding of STATTIC has shown to contribute to its potent inhibitory activity toward STAT3 (51). In contrast, the larger two compounds C01L_F03 and STX-0119 covered both pTyr-binding and hydrophobic SH2 cavities for binding, at the same time. Not surprisingly, the non-specific *in silico* binding of STATTIC and STX-0119 toward all STATs (STAT1-STAT6) (16), could also be observed for C01L_F03 (data not shown). Together our data provide a molecular basis for STAT-cross-binding specificity of C01L_F03, STATTIC, and STX-0119 and their potential to inhibit multi-STAT-activity and target gene expression.

In general STAT direct inhibitors are a very heterogenous group considering their chemical attributes e.g., peptides, peptidomimetics, natural and synthetic small compounds. In our work we concentrated on small compounds targeting STAT-STAT dimerization plane between pTyr linker and SH2 domain, with pTyr-binding cavity as a main interaction site. Since interaction between negatively charged pTyr residue and positively charged Arg and Lys in the cavity is very relevant for STAT-STAT dimerization, the main driving force of the binding are polar and electrostatic interactions. Thus, we postulate that the general common chemical attribute of STAT inhibitors might be negatively charged side group analogous to pTyr phosphate (which is common for the tested compounds). Our results are in agreement with other studies on the binding mode of STAT inhibitors, e.g., Arpin et al. (52) who reported a novel STAT3 small molecule inhibitor PG-S3-001 as a pancreatic cancer therapeutic. They performed detailed *in silico* characteristics of its binding to the STAT3-SH2 domain. Similar to their results, in our docking studies STATTIC, STX-0119, and C01L_F03 targeted the pTyr-binding cavity and exhibited polar and electrostatic interactions between negatively charged side groups and the same amino acids as reported by Arpin et al. (52) For example, PG-S3-001 binds with Arg609, Lys591 and Ser611 by carboxyl group, whereas STATTIC interacts with the same amino acids in STAT3 by the nitro group and the corresponding amino acids in STAT1 and STAT2. Moreover, STX-0119 and C01L_F03 possess a relatively flexible glycine core similarly to PG-S3-001.

The comparative docking simulations and *in vitro* inhibition studies related to STAT-cross-binding specificity correspond to other studies. For example, Bill et al. provided evidence for cross-binding of curcumin to STAT3 and STAT1 (53). Other natural products like cryptotanshinone (54) and resveratrol analogs (RSVA314 and RSVA405) (55) exhibited similar characteristics. Sanseverino et al. found that STATTIC inhibits not only STAT3 activation but also that of STAT1 and to a lesser extent of STAT2, in response to cell activation by IL-6 or IFNβ based on studies using human monocyte-derived dendritic cells (56). This correlates with our finding, that STATTIC is not STAT3 specific. Inhibition of STAT1 phosphorylation by STATTIC has also been described in human ovarian cancer cells (57) and melanoma cells (58). Therefore, evidence accumulates that many of the known STAT3 inhibitors do not seem STAT3 specific.

Docking simulations of C01L_F03, STATTIC, and STX-0119 in combination with *in silico* STAT-SH2 mutagenesis provided further evidence to suggest that these compounds directly interact with hSTAT1, hSTAT2, and hSTAT3 (Figure 6). In this respect, docking simulations highly correlated with *in vitro* mutagenesis studies of a.a. R602 in STAT1, R601 in STAT2 and R609 in STAT3, which were experimentally proven to be crucial for STAT phosphorylation and reciprocal binding of the pTyr-linker to the STAT-SH2 domain [STAT1-R602 (45); STAT2-R601 (46); STAT3-R609 (47)]. The same was true, for the second mutation a.a. K584 in STAT1 (48), R583 in STAT2 and K591 in STAT3 (18). In case of STATTIC and its selective binding to STAT3, the lower STAT3 binding affinity of published STATTIC analogs, STB and STC (13)] coincided with decreased binding stability toward STAT-SH2 models (Figure 6). Combined with the observed *in vitro* effects of C01L_F03 on STAT DNA-binding (Figure 3) this strongly suggests that all three compounds act as direct STAT-inhibitors. This is also in line with the finding that the activation state of the tyrosine kinases JAK1, JAK2, and c-Src, which are considered to be responsible for phosphorylation of STAT3 Tyr705, was not significantly inhibited by the presence of 10 or 20 mM STATTIC in breast cancer cells (13).

Of all STATs especially STAT1, STAT2, and STAT3 have been recognized as prominent modulators of inflammation, especially in immune and vascular cells during atherosclerosis (7, 59). However, STAT-inhibitory strategies targeting CVDs, still await entering the clinic. Based on the newly identified STAT cross-binding mechanism for C01L_F03, STATTIC, and STX-0119, we subsequently pursued a multi-STAT inhibitory approach as a novel strategy in the treatment of vascular inflammation and CVDs. Along these lines, we first tested the effect of C01L_F03, STATTIC, and STX-0119 on signal integration between IFNγ and LPS, which in vascular cells and atheroma interacting immune cells modulates important aspects of vascular inflammation (10) (Table 6). Indeed, pre-treatment of ECs with C01L_F03, STATTIC, or STX-0119 resulted in a similar inhibition pattern of IFNγ+LPS induced expression of the genes IFIT2, OAS2, CCL5, CXCL10, CXCL9, ICAM1, and VCAM1, with STATTIC being the most potent one (Figure 7). Likewise, STATTIC potently inhibited expression of the pro-inflammatory and pro-atherogenic genes CXCL9, CXCL10, CCL5, Nos2, IFIT1, and OAS2 in VSMCs treated with IFNγ and LPS (not shown). This suggested that C01L_F03, STATTIC, and STX-0119 may commonly block STAT cooperative promotor activation with IRF and NF-κB mediated by IFNγ and LPS in ECs and VSMCs. To provide further evidence for this, we decided to study the genome-wide effect of C01L_F03, STATTIC and STX-0119 on IFNγ+LPS-mediated vascular inflammation (Figure 8). Thus, IFNγ+LPS increased the expression of 731 genes, of which 159 were commonly inhibited by C01L_F03, STATTIC, and STX-0119. These 159 genes generally represented the ones with the highest IFNγ+LPS inducible levels and their biological functions reflected strong inhibitory potential of C01L_F03, STATTIC, and STX-0119 toward pro-inflammatory and proatherogenic responses. Among those genes many known STAT target genes (i.e., SOCS1, IRF1, IRF8, APOL1, BID as STAT1 targets; IFIT1, IFIT2, IFIT3, OAS1, OAS2, MX1, MX2, ISG15 as STAT1-STAT2 targets; SOCS3, CCND1, MMP3, FAS PIM1, VEGF, S1PR1 as STAT3 targets) could be recognized. More important, promoter analysis of the 159 commonly inhibited genes, for the presence of ISRE, STAT or NF-κB binding sites provided additional evidence that C01L_F03, STATTIC, and STX-0119 are “multi-STAT” as well as “STAT-only” inhibitors that commonly inhibit pro-inflammatory and pro-atherogenic gene expression directed by cooperative involvement of multiple STATs with IRFs and/or NF-κB.

**Table 6:** Comparison of top-12 GO terms, selected based on Fold enrichment values, between genes induced by treatment with IFNγ+LPS and group of IFNγ+LPS induced and inhibited simultaneously by C01L_F03, STATTIC, and STX-0119.

**GO Term**	**Biological process**	**Induced by IFN**γ **and LPS**			**Inhibited by C01L_F03, STATTIC and STX-0119**		
		**Fold enrichment**	**Uniqueness**	**Dispensability**	**Fold enrichment**	**Uniqueness**	**Dispensability**
GO:0043207	Response to external biotic stimulus	55.28	0.84	0.00	28.14	0.82	0.11
GO:0009607	Response to biotic stimulus	54.84	0.92	0.12	27.38	0.92	0.12
GO:0006952	Defense response	52.46	0.88	0.43	29.61	0.88	0.43
GO:0019221	Cytokine-mediated signaling pathway	52.09	0.74	0.11	29.62	0.76	0.00
GO:0002376	Immune system process	49.72	1.00	0.00	28.66	0.99	0.00
GO:0001817	Regulation of cytokine production	37.65	0.83	0.00	10.67	0.81	0.05
GO:0007166	Cell surface receptor signaling pathway	28.05	0.79	0.28	13.89	0.81	0.28
GO:0006954	Inflammatory response	27.36	0.86	0.68	8.28	0.87	0.68
GO:0042127	Regulation of cell proliferation	23.04	0.86	0.71	7.97	0.87	0.71
GO:0042981	Regulation of apoptotic process	22.99	0.82	0.83	4.23	0.86	0.83
GO:0030334	Regulation of cell migration	17.90	0.81	0.05	4.70	0.72	0.76
GO:0030155	Regulation of cell adhesion	15.23	0.88	0.05	9.09	0.86	0.05

Based on the previous studies, transcription of genes containing STAT-, ISRE- and NF-κB-binding sites in their promoter regions are under the cooperative regulation by extracellular stimuli activating STATs, IRFs and NF-κB, such as IFNγ, IFNα and TNFα, IL-1β or LPS (60–68). In general it is believed that in immune cells, but also in vascular cells, multiple inflammatory stimuli culminate in gene expression that requires cooperation of STATs with IRFs and/or NF-κB (69). They are responsible for promoting type I immune actions associated with host-defense mechanisms against viral and bacterial infections and excessive immune responses (70) at the basis of different diseases, including CVDs. This correlates with our recent data mining studies of atherosclerotic plaque transcriptomes. In this study we performed detailed promoter analysis of differentially expressed inflammatory genes in coronary and carotid plaques and predicted cooperative involvement of NF-κB, STATs, and IRFs (on ISRE, GAS, ISRE/GAS, ISRE/NF-κB, or GAS/NF-κB binding sites) (71). Combined with our findings here, this suggests strong inhibitory potential of C01L_F03, STATTIC, and STX-0119 toward vascular inflammation and vascular dysfunction.

The fact that among the 159 genes that were commonly inhibited by C01L_F03, STATTIC and STX-0119 were multiple chemokines and adhesion molecules, prompted us to investigate the effect of a multi-STAT inhibitory strategy on IFNγ+LPS dependent ECs migration and leukocyte adhesion to ECs. The endothelial scratch wound (migration) assay has been described as a simple and well-developed method to measure cell migration *in vitro* (37), which reflects vascular and immune cell migration during atherosclerosis. In addition, pathological angiogenesis of the vessel wall is a consistent feature of atherosclerotic plaque development and progression of the disease (72). Indeed, a significant decrease in IFNγ+LPS-induced “wound healing” of scratched ECs could be detected in the presence of C01L_F03, STATTIC, and STX-0119 (Figure 9). Interestingly a subset of C01L_F03, STATTIC and STX-0119 inhibited chemokines, including CXCL9, CXCL10, CCL7, CCL8, CCL3L3, CCL5, and CCRL2 (Table 5), has been reported to be increased in cells from the vasculature. Also, transcriptional regulation of a number of these genes in response to IFNγ and LPS in various cell types was shown to involve multiple STATs, IRFs and or NF-κB (10, 71). This coincides with our results here, but also with our recently published data, in which elevated expression of the chemokines CXCL9 and CXCL10 mirrored pSTAT1 levels in VSMCs and ECs of human atherosclerotic plaques (10). Moreover, it was proved that chemokines cooperate in leukocyte recruitment to the injured artery during vascular remodeling (73–75) and as such are involved in the pathogenesis of atherosclerosis. Our observation that C01L_F03, STATTIC, and STX-0119 were also able to significantly inhibit IFNγ-and LPS-dependent expression of VCAM1 and ICAM1 (Figures 7, **8**, Table 5, Table S1) as well as dramatically reduce adhesion of leukocytes to ECs under dynamic flow conditions (Figure 10), is in line with a prominent role for both adhesion molecules in these phenomena (59). Moreover, the transcriptional regulation of both ICAM1 and VCAM1 has shown to depend on several transcription factors, including multiple STATs, IRFs, and NF-κB (59, 62, 76). This could provide an explanation for the potent inhibitory effect of C01L_F03, STATTIC, and STX-0119 on IFNγ+LPS-induced adhesion of leukocytes to ECs, however we cannot exclude the possibility that other adhesion molecules may also be involved.

Finally, a multi-STAT inhibitory strategy was tested for the potential to inhibit IFNγ+LPS induced impairment of mesenteric artery contractility (Figure 11). Previously, we observed that the signal integration between IFNγ and LPS in mesenteric artery segments resulted in impaired contractility (Figure 11A). This finding overlapped with a dramatic increase in VSMC-specific expression of Nos2 (10), which is associated with progression of atherosclerosis by participating in vascular dysfunction (77, 78). Now we prove for the first time that C01L_F03, STATTIC, and STX-0119 are able to protect against IFNγ and LPS induced impairment of mesenteric artery contractility, likely by inhibiting Nos2 expression. The transcriptional regulation of Nos2 in response to IFNγ and LPS also has shown to depend on several transcription factors, including STATs, IRFs, and NF-κB (61, 79).

STATTIC as well as STX-0119 have shown to increase the apoptotic rate of a variety of cancer cell lines *in vitro* and in tumors *in vivo*, in a STAT3-dependent manner. In our studies, STATTIC and STX-0119, but also C01L_F03 exhibited cytotoxic effects at the highest used concentrations. It is possible that this cell death is mediated by inhibiting the anti-apoptotic effects of STAT3. However, at lower concentrations at which all three inhibitors potently inhibited STAT-dependent pro-inflammatory and pro-atherogenic gene expression, this cell death was not visible. Surprisingly, in the mesenteric artery contractility experiments STATTIC and STX-0119 could only be used in the nM range, without causing IFNγ+LPS-independent impairment of vessel function and integrity. This is a thousand fold less as in the wound healing and adhesion assay and could point to a greater sensitivity of STATTIC and STX-0119 *in vivo* as compared to *in vitro*.

In agreement with literature, targeting the STAT3 pathway is an upcoming therapeutic approach in the treatment of a rising number of inflammatory or proliferative diseases, like myelofibrosis, myeloproliferative disorders, rheumatoid arthritis and colitis ulcerosa also has a modulating effect on vascular cell function. Several FDA-approved indirect STAT3 inhibitors (Ruxolitinib: JAK1/2-inhibition; Tocilizumab: IL-6 receptor antibody; Tofacitinib: pan-JAK inhibition) as well as currently tested known drugs in clinical trials for CVDs treatment (Sirukumab: IL-6 binding antibody; Baricitinib: JAK1/JAK2 inhibitor), predict the use of STAT3-inhibiting clinical strategies in the near future (80). Recently, Johnson et al. for the first time showed that STATTIC and S3I-201 protect against AngII-induced oxidative stress, endothelial dysfunction, and hypertension in mice (81). Because AngII promotes vascular disease in the presence of multiple cardiovascular risk factors, the authors suggested that selective targeting of STAT3 might have substantial therapeutic potential. However, as S3I-201 and STATTIC are not STAT3-specific (16), an additional role of other STATs like STAT1 cannot be ruled out (82–85).

A large number of independent studies confirm the potency of STATTIC as a direct STAT3 inhibitor and support its utility in combating tumor cells. These studies demonstrate the potent anticancer activities of STATTIC, including activity against glioma cell migration on three-dimensional nanofiber scaffolds (86), colon cancer-initiating cells (87), and against outgrowth of breast cancer cells in an *ex vivo* model (88), and extend to *in vivo* activity of STATTIC in a mouse xenograft model for head and neck squamous cell carcinoma (89). Likewise, STX-0119 also demonstrated potent antitumor effects *in vivo* in SCC3-bearing nude mice in a STAT3-dependent manner (14). With their ability to function as multi-STAT inhibitors, like C01L_F03, they could additionally act as potent inhibitors of vascular inflammation in atherosclerosis.

In conclusion, our STAT-inhibitory studies of C01L_F03, STATTIC and STX-0119 and our previous revelation of STAT cross-binding of a pre-selection of known STAT3 inhibitors in combination with the literature, collectively provide evidence for a novel class of multi-STAT inhibitory compounds that target cooperative involvement of multiple STATs with NF-κB and/or IRFs (on ISRE, GAS, ISRE/GAS, ISRE/NF-κB, or GAS/NF-κB binding sites) in the regulation of crucial pro-inflammatory and pro-atherogenic target genes (7). Based on this we propose their potential as a potent clinical application in CVDs apart from their established role in cancer treatment and prevention. It should be noted that the primary aim of our study was to test our novel comparative *in silico* docking STAT-inhibitor selection strategy and offer support for the possibility of using a multi-STAT inhibitory approach in the context of vascular inflammation. However, our future studies will be dedicated to optimize our selection strategy and identify new STAT inhibitors with higher potency and bioavailability. Further testing and optimizing of already available non-specific STAT inhibitors like STATTIC, i.e., by chemical modification, may also be a promising avenue. In this respect it is also important to consider that STATs are essential factors to maintain normal homeostasis in many body organs and tissues. Consequently, for the treatment of single atherosclerotic lesions and to prevent systemic effects on other STATs, a local, “targeted” application with negligible systemic side effects might be a favorable scenario. Finally, it is important to emphasize that IL-10 and IL-6 produced by macrophages in the atherosclerotic plaque both regulate STAT3, yet generate different cellular responses. IL-6 is primarily a pro-inflammatory cytokine, whereas IL-10 generates a strong anti-inflammatory response. IL-6 and IL-10 each bind to their cognate receptor, leading to STAT3 phosphorylation, nuclear localization, and a cytokine-specific gene activation pattern. Thus, within the same cell type STAT3 can be pro- and anti-inflammatory (90, 91). When responding to certain stimuli such as inflammatory mediators or microbial products, macrophages have the ability to be polarized into M1 and M2 subtypes. M1 macrophages express low levels of IL-10, M2 macrophages express abundant IL-10 and can both be detected in atherosclerotic lesions. A macrophage phenotypic switch from M2 to M1 occurs with lesion progression and M1 macrophages dominate over M2 macrophages in the rupture-prone shoulder regions of the plaque, whereas M2 polarized cells are found in stable plaques (91). Current strategies of STAT3 inhibition do not consider IL-10 action. Thus, for the anti-atherosclerotic treatment with Statinibs to be most effective a treatment strategy should be considered during the stages were the M1 phenotype is dominant over the M2 phenotype, which correlates with low IL-10 levels and during which activation of STAT3 by IL-6 shifts to pro-inflammatory responses.

## Author contributions

All the authors significantly contributed to research and to experimental process. Under the supervision of HB, MS performed *in silico* docking of STAT-SH2 models and MP-G performed *in vitro* STAT inhibition validation. Both were also involved in statistical analysis, microarray data analysis and manuscript preparation. JW assisted in microarray experiments and counseled during the process of data analysis. AC, PM, and MJS from the University of Valencia performed leukocyte adhesion experiments. SV, MR-G, and CP from Universidad Autónoma de Madrid performed *ex vivo* contractility studies. Both groups, from Valencia and Madrid were also involved in manuscript preparation/writing process.

### Conflict of interest statement

The authors declare that the research was conducted in the absence of any commercial or financial relationships that could be construed as a potential conflict of interest.

## Abbreviations

ACTB: Actin Beta
APOL1: Apolipoprotein L1
BCA: Bicinchoninic acid
BID: BH3 Interacting Domain Death Agonist
BS: Binding Score value
CAVS: Comparative Approach for Virtual Screening
CBAV: Comparative Binding Affinity Value
CCL3L3: C-C Motif Chemokine Ligand 3 Like 3
CCL5: C-C Motif Chemokine Ligand 5
CCL7: C-C Motif Chemokine Ligand 7
CCL8: C-C Motif Chemokine Ligand 8
CCND1: Cyclin D1
CD74: Cluster of Differentiation 74
CDL: Clean Drug-Like
CL: Clean Leads
CVDs: Cardiovascular Diseases
CXCL10: C-X-C motif chemokine 10
CXCL9: C-X-C motif chemokine 9
ECs: Endothelial Cells
EGF: Endothelial Growth Factor
FAS: Fas Cell Surface Death Receptor
FBS: Fetal Bovine Serum
FC: Fold Change
GAS: Interferon-Gamma Activated Sequence
GBP4: Guanylate Binding Protein 4
GBP5: Guanylate Binding Protein 5
GO: Gene Ontology
HMECs: Human Microvascular Endothelial Cells
HRP: Horseradish Peroxidase
HUVECs: Human Umbilical Vein Endothelial Cells
ICAM1: Intercellular Adhesion Molecule 1
IFI44L: Interferon Induced Protein 44 Like
IFIT1: Interferon Induced Protein with Tetratricopeptide Repeats 1
IFIT2: Interferon Induced Protein with Tetratricopeptide Repeats 2
IFIT3: Interferon Induced Protein with Tetratricopeptide Repeats 3
IFN: Interferon
INDO, Indoleamine 2: 3-Dioxygenase 1
IRF: Interferon Regulatory Factor
IRF1: Interferon Regulatory Factor 1
IRF8: Interferon Regulatory Factor 8
ISG15: Interferon-Stimulated Gene 15
ISRE: Interferon-Stimulated Response Element
LBPV: Ligand Binding Pose Variation
LPS: Lipopolysaccharide
MMP12: Matrix Metalloproteinase-12
MMP3: Matrix Metalloproteinase-3
NF-κB: Nuclear Factor κB
OAS1: 2′-5′-Oligoadenylate Synthetase 1
OAS2: 2′-5′-Oligoadenylate Synthetase 2
PBS: Phosphate Buffered Saline
PIM1: Pim-1 Proto-Oncogene
S1PR1: Sphingosine-1-Phosphate Receptor 1
SCC3: Sister-Chromatid Cohesion protein 3
SH2: Src Homology 2 Domain
SOCS3: Suppressor of Cytokine Signaling 3
STAT: Signal Transducer and Activator of Transcription
TLR4: Toll-like Receptor 4
TNF: Tumor Necrosis Factor
UBD: Ubiquitin D
VCAM1: Vascular Cell Adhesion Molecule 1
VEGF: Vascular Endothelial Growth Factor
VSMCs: Vascular Smooth Muscle Cells.
