# Supplementary material for: Genome-Wide Inhibition of Pro-atherogenic Gene Expression by Multi-STAT Targeting Compounds as a Novel Treatment Strategy of CVDs
Source: Front Immunol. 2018 Sep 19;9:2141. doi: 10.3389/fimmu.2018.02141 (PMC6156247; doi:10.3389/fimmu.2018.02141)
Supplement: Supplementary file 1 [file Data_Sheet_1.docx]

**Figure S1**

**
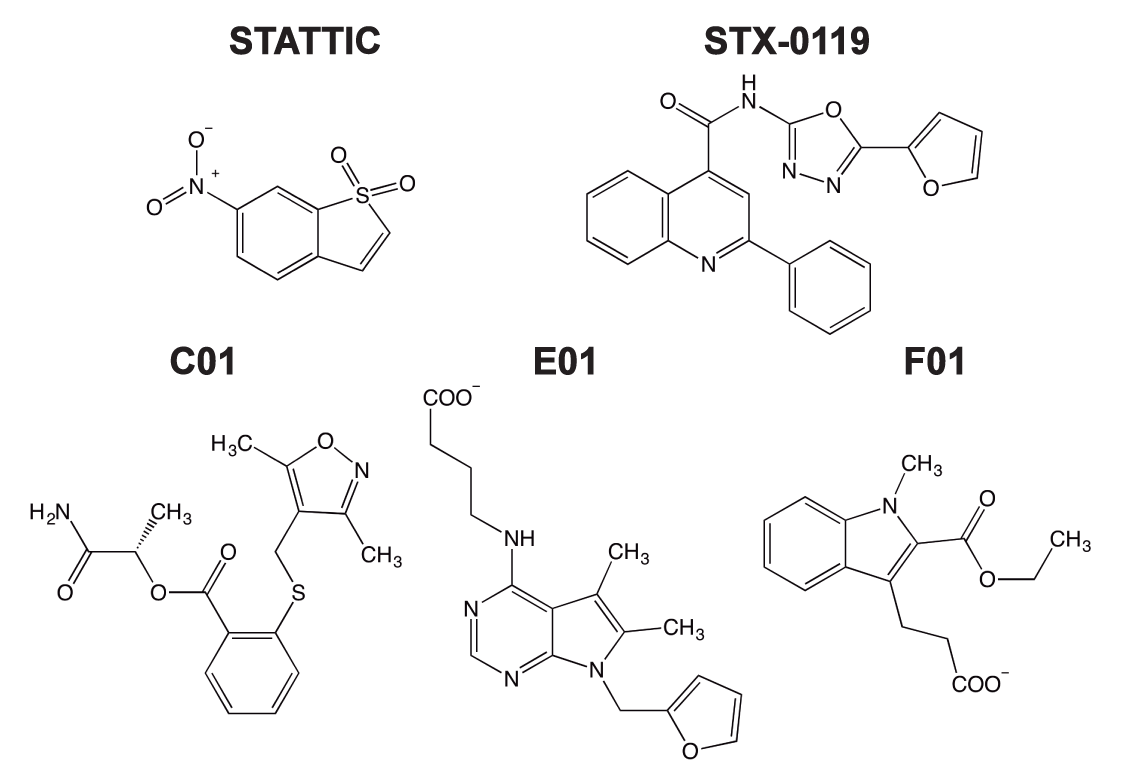
**

**Geometries of tested STAT3 inhibitors (STATTIC and STX-0119) and compounds selected from Clean Leads after primary screen (C01, E01 and F01)**.

**Figure S2**

**
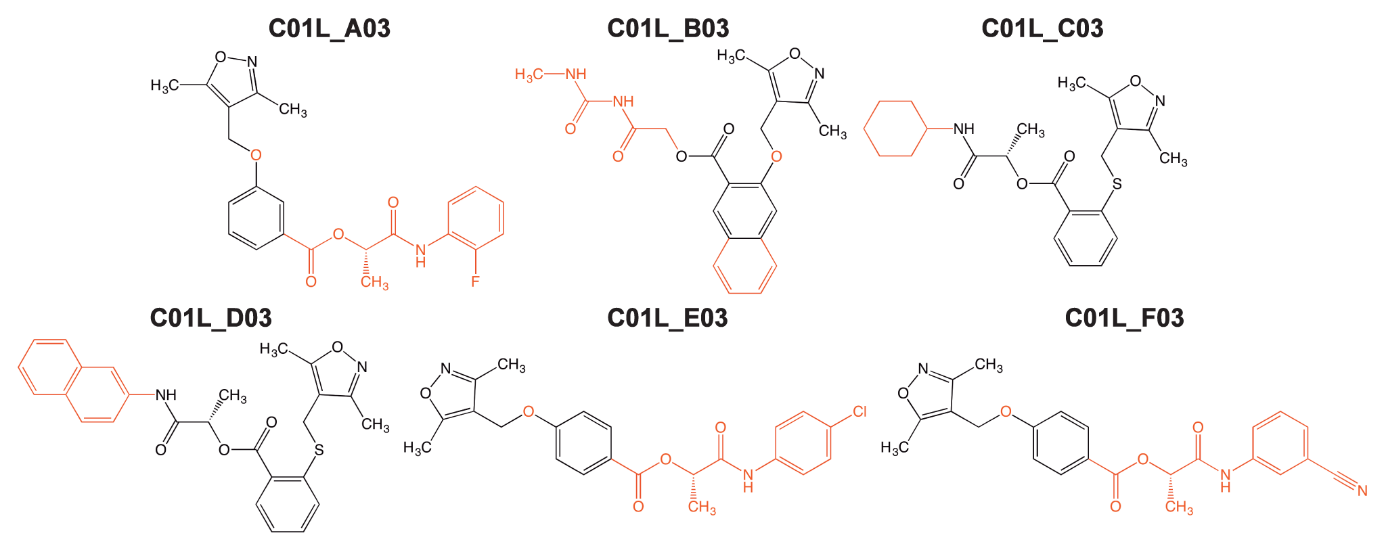
**

**Geometries of top 6 C01-like compounds selected after secondary screen from ZINC Database.** Red color indicates structural differences in atomic backbone between C01 and C01-like compounds.

**Figure S3**


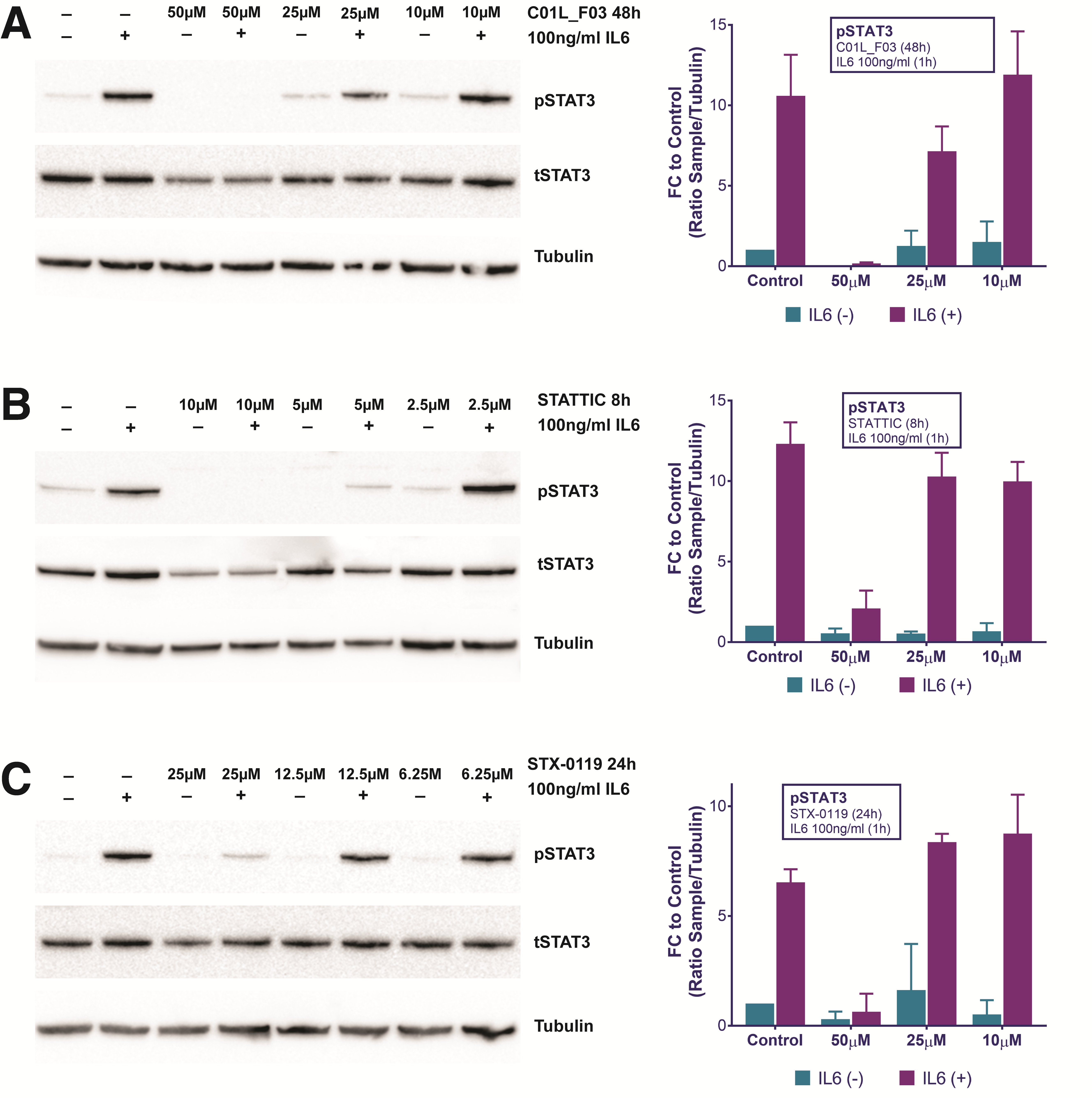


**C01L_F03, STATTIC and STX-0119 inhibit IL6-induced STAT3 phosphorylation.** HMECs were treated with: 50µM, 25µM, 10µM and 5µM C01L_F03 for 48h **(A)**, 10µM, 5µM, 2.5µM of STATTIC for 8h **(B)** or with 25µM, 12.5µM, 6.25µM of STX-0119 for 24h **(C)** and with 100ng/ml of IL6 for 1h. Protein extracts were collected and levels of pSTAT3, tSTAT3 and α-tubulin were assessed by western blotting. Western quantification **(A, B, C)**. Bars represent mean quantification form 3 individual repeats ± SEM as error bars.

**Figure S4**


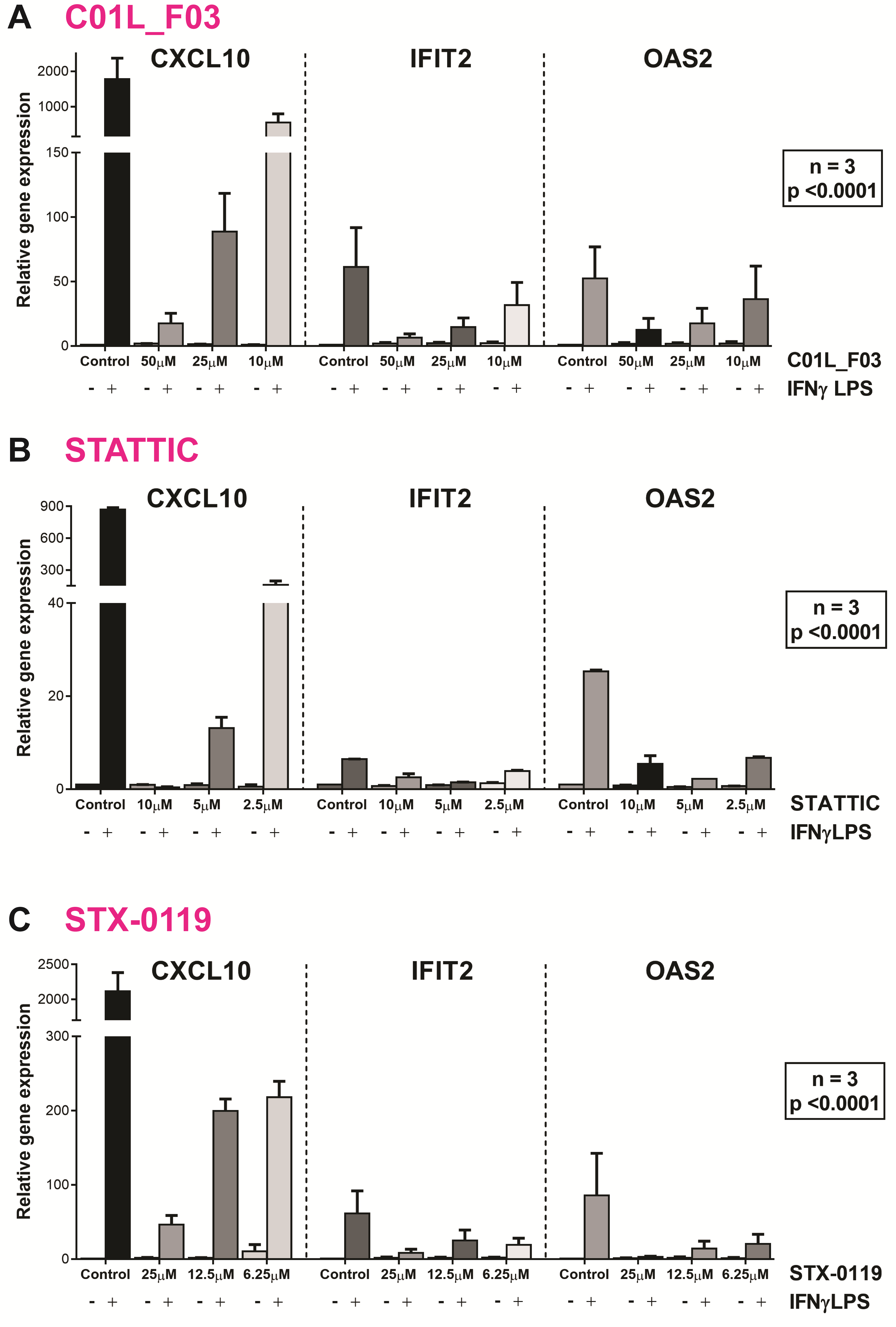


**C01L_F03 (A), STATTIC (B) and STX-0119 (C) inhibit IFNγ+LPS induced gene expression of CXCL10, IFIT2 and OAS2 in therapeutic mode treatment.** HMECs were treated with **(A)** 50µM, 25µM, 10µM of C01L_F03 for 24h or **(B)** 10µM, 5µM, 2.5µM of STATTIC for 6h or **(C)** with 25µM, 12.5µM, 6.25µM of STX-0119 for 24h and for 24h with IFNγ + 8h or 4h with LPS. RNA was isolated and subjected to qPCR analysis. Experiments were performed in 3 individual repeats, which were compared by two-way ANOVA test and unpaired two-tailed student T-test.

**Table S1**

**The complete list of up-regulated genes in response to IFNγ+LPS in the presence or absence of C01L_F03, STATTIC and STX-0119.** The table shows genes induced at least 2 times, in comparison to untreated cells. List was obtained by raw data analysis from microarray experiment using Standard Illumina Expression BeadChip HumanHT-12v4. Additionally, we show fold change values in cells which were treated with: 50µM of C01L_F03, 25µM of STX-0119 or 10µM of STATTIC in presence of IFNγ+LPS. Treatment with IFNγ+LPS resulted in increased expression of 730 genes which are listed in descending order.

| **Gene ID** | **Fold change relative to control** | | | |
| --- | --- | --- | --- | --- |
|  | **IFNγ+LPS** | **C01L_F03** | **STATTIC** | **STX-0119** |
| **CXCL10** | 9298.61 | 615.79 | 223.90 | 15.14 |
| **CCL8** | 2118.86 | 6.83 | 0.79 | 6.31 |
| **UBD** | 1860.83 | 129.28 | 62.19 | 2.09 |
| **CXCL9** | 1565.00 | 2.26 | 6.31 | 1.00 |
| **GBP4** | 793.16 | 38.79 | 8.61 | 16.30 |
| **CCL20** | 529.63 | 207.11 | 112.86 | 0.79 |
| **GBP5** | 435.89 | 20.26 | 11.63 | 5.26 |
| **CSF2** | 352.98 | 43.55 | 93.02 | 3.62 |
| **OAS2** | 329.65 | 2.61 | 1.48 | 6.29 |
| **VCAM1** | 326.83 | 33.01 | 8.12 | 1.36 |
| **CCL7** | 289.01 | 1.00 | 1.00 | 3.41 |
| **INDO** | 209.33 | 1.00 | 1.00 | 1.80 |
| **MMP3** | 192.62 | 2.22 | 2.89 | 28.96 |
| **LINCR** | 191.82 | 9.19 | 20.68 | 1.81 |
| **IDO1** | 178.80 | 1.62 | 1.42 | 6.03 |
| **TNF** | 177.70 | 57.91 | 40.78 | 1.00 |
| **CCL3L3** | 165.18 | 4.69 | 20.25 | 1.52 |
| **OASL** | 160.22 | 3.99 | 14.16 | 1.21 |
| **CXCL2** | 144.97 | 325.68 | 257.65 | 2.76 |
| **LYPD5** | 127.07 | 6.75 | 7.66 | 6.90 |
| **LTB** | 125.33 | 1.00 | 0.95 | 0.75 |
| **MMP12** | 111.79 | 0.70 | 0.70 | 0.70 |
| **IL6** | 100.46 | 21.53 | 28.10 | 3.94 |
| **IFI44L** | 100.04 | 1.14 | 1.21 | 1.14 |
| **TNFAIP3** | 99.30 | 65.87 | 183.81 | 2.76 |
| **LOC730249** | 91.23 | 0.60 | 0.78 | 0.96 |
| **CD74** | 88.67 | 1.18 | 0.84 | 0.76 |
| **CCL5** | 83.48 | 3.77 | 25.58 | 1.08 |
| **IFNB1** | 77.51 | 13.57 | 388.03 | 1.00 |
| **LOC100129681** | 76.64 | 1.31 | 0.18 | 2.35 |
| **MX2** | 68.99 | 0.40 | 1.23 | 0.74 |
| **DLL1** | 68.78 | 3.39 | 8.11 | 10.80 |
| **IL8** | 64.98 | 51.02 | 44.10 | 8.51 |
| **LOC400759** | 64.91 | 16.78 | 8.77 | 6.48 |
| **C1S** | 64.57 | 4.22 | 2.39 | 5.98 |
| **TNFSF10** | 64.45 | 0.67 | 0.21 | 1.22 |
| **HAPLN3** | 63.96 | 9.15 | 3.93 | 8.98 |
| **IL1A** | 62.96 | 16.26 | 34.11 | 1.73 |
| **RSAD2** | 62.08 | 2.04 | 1.58 | 1.21 |
| **XIRP1** | 61.99 | 1.00 | 1.00 | 1.00 |
| **HLA-DOB** | 61.70 | 1.00 | 1.07 | 1.75 |
| **BST2** | 59.70 | 1.83 | 0.77 | 2.06 |
| **GBP2** | 58.72 | 12.47 | 5.53 | 10.10 |
| **IL18BP** | 58.65 | 9.10 | 2.69 | 12.59 |
| **IFIT3** | 57.94 | 3.63 | 8.58 | 2.78 |
| **CXCL6** | 56.71 | 4.37 | 0.75 | 1.04 |
| **CXCL1** | 56.42 | 39.85 | 31.30 | 1.28 |
| **TRAF1** | 56.16 | 2.73 | 3.49 | 1.06 |
| **TNFSF18** | 55.06 | 0.85 | 3.99 | 3.16 |
| **MX1** | 53.42 | 2.10 | 1.36 | 1.95 |
| **TNFAIP2** | 51.66 | 5.74 | 2.38 | 0.70 |
| **ICAM1** | 50.66 | 29.16 | 7.84 | 6.47 |
| **IFIH1** | 50.41 | 4.21 | 4.11 | 1.14 |
| **IFIT2** | 50.17 | 3.40 | 12.29 | 2.98 |
| **PLA1A** | 49.40 | 0.72 | 1.11 | 0.72 |
| **IFI44** | 49.39 | 1.09 | 2.45 | 1.14 |
| **C15orf48** | 48.03 | 1.27 | 0.97 | 1.87 |
| **TMEM171** | 47.65 | 2.26 | 2.40 | 1.63 |
| **IFITM1** | 46.56 | 1.97 | 1.15 | 2.30 |
| **IL1B** | 45.93 | 7.73 | 6.22 | 1.26 |
| **LOC400696** | 43.45 | 1.00 | 1.00 | 0.83 |
| **CMPK2** | 42.80 | 2.17 | 1.30 | 1.60 |
| **LOC285016** | 42.74 | 8.25 | 2.03 | 1.70 |
| **BDKRB1** | 41.68 | 1.00 | 2.23 | 2.39 |
| **LMO2** | 41.15 | 1.06 | 0.38 | 1.57 |
| **ZC3H12A** | 38.92 | 21.99 | 28.93 | 8.14 |
| **BATF2** | 38.20 | 4.64 | 1.17 | 6.77 |
| **IRG1** | 37.77 | 0.62 | 0.62 | 0.62 |
| **IRF8** | 37.73 | 12.15 | 5.79 | 17.93 |
| **LOC100128274** | 35.83 | 3.87 | 1.35 | 1.11 |
| **TNFSF13B** | 35.63 | 2.50 | 2.52 | 1.57 |
| **MIR155HG** | 34.60 | 5.91 | 3.92 | 0.81 |
| **SAMD9L** | 34.25 | 2.64 | 0.99 | 1.85 |
| **ETV7** | 34.12 | 8.71 | 2.33 | 5.05 |
| **FBXO6** | 32.86 | 8.80 | 7.09 | 4.09 |
| **OAS1** | 32.58 | 1.62 | 0.90 | 1.20 |
| **HERC5** | 31.81 | 1.21 | 1.60 | 1.30 |
| **SOCS1** | 30.83 | 2.89 | 1.95 | 4.07 |
| **DHX58** | 30.58 | 3.17 | 4.22 | 1.51 |
| **FAM46A** | 30.19 | 1.85 | 5.38 | 12.46 |
| **CCL3L1** | 29.53 | 0.36 | 4.18 | 1.61 |
| **LGALS9** | 29.30 | 1.00 | 1.00 | 1.00 |
| **CFB** | 28.41 | 2.40 | 1.41 | 1.46 |
| **NOD2** | 28.11 | 3.64 | 2.55 | 1.95 |
| **IL15RA** | 28.04 | 5.26 | 1.31 | 2.84 |
| **LIF** | 27.83 | 2.92 | 3.29 | 1.31 |
| **G0S2** | 27.24 | 5.64 | 4.06 | 1.39 |
| **FST** | 25.71 | 0.49 | 0.58 | 2.18 |
| **PDLIM4** | 25.17 | 1.93 | 1.49 | 0.79 |
| **DDX58** | 25.10 | 2.08 | 0.91 | 1.39 |
| **NLF2** | 24.47 | 11.26 | 2.79 | 3.60 |
| **CIITA** | 24.37 | 1.17 | 1.32 | 0.42 |
| **GPR37L1** | 23.55 | 1.00 | 1.85 | 1.00 |
| **KRT16** | 23.44 | 10.27 | 2.56 | 2.50 |
| **TRIM22** | 23.29 | 0.87 | 1.08 | 2.44 |
| **APOL1** | 22.60 | 2.39 | 2.54 | 1.85 |
| **APOL6** | 22.57 | 5.77 | 4.67 | 3.52 |
| **USP18** | 21.74 | 0.39 | 0.73 | 0.98 |
| **PTX3** | 21.61 | 6.96 | 5.95 | 1.03 |
| **MIR1275** | 21.40 | 12.20 | 11.18 | 3.35 |
| **RTP4** | 19.88 | 0.40 | 1.17 | 0.82 |
| **ARID5A** | 19.80 | 2.33 | 7.80 | 6.03 |
| **SOD2** | 19.57 | 3.65 | 3.71 | 1.88 |
| **XAF1** | 19.37 | 1.63 | 1.52 | 1.11 |
| **KLF4** | 19.13 | 5.54 | 8.37 | 6.11 |
| **SERPING1** | 18.85 | 2.55 | 4.97 | 3.16 |
| **LOC728835** | 18.66 | 1.00 | 1.17 | 1.00 |
| **C8orf4** | 18.41 | 7.02 | 2.27 | 1.27 |
| **TNFRSF9** | 18.31 | 8.03 | 3.42 | 2.09 |
| **WARS** | 17.64 | 10.02 | 4.42 | 2.80 |
| **MIR302C** | 17.25 | 9.98 | 8.25 | 1.17 |
| **LOC729231** | 17.10 | 4.71 | 4.95 | 11.38 |
| **CD38** | 16.88 | 2.10 | 1.93 | 0.46 |
| **HSH2D** | 16.55 | 1.00 | 1.00 | 1.00 |
| **GCH1** | 16.38 | 2.18 | 1.67 | 1.30 |
| **IL32** | 16.13 | 3.80 | 2.64 | 2.06 |
| **IFI27** | 16.11 | 3.21 | 1.49 | 1.86 |
| **IFIT1** | 15.95 | 0.60 | 1.35 | 0.58 |
| **GBP1** | 14.94 | 7.38 | 5.26 | 5.26 |
| **PDCD1LG2** | 14.89 | 2.42 | 2.20 | 3.39 |
| **TP73L** | 14.63 | 0.11 | 0.30 | 0.36 |
| **BIRC3** | 14.52 | 5.65 | 5.08 | 1.10 |
| **C2CD4B** | 14.35 | 9.00 | 1.57 | 3.11 |
| **LOC390975** | 14.24 | 1.00 | 1.59 | 5.56 |
| **RARRES3** | 14.18 | 2.15 | 0.96 | 2.54 |
| **C1R** | 14.14 | 1.87 | 1.63 | 1.60 |
| **LOC653879** | 13.98 | 3.13 | 2.68 | 2.01 |
| **LOC285047** | 13.87 | 0.57 | 0.57 | 1.57 |
| **CCL2** | 13.58 | 2.00 | 1.09 | 2.65 |
| **IFI30** | 13.40 | 2.20 | 0.46 | 2.04 |
| **LOC100130696** | 12.22 | 7.11 | 11.70 | 8.07 |
| **STARD8** | 12.03 | 2.72 | 1.44 | 1.84 |
| **ISG20** | 11.92 | 1.42 | 2.33 | 1.60 |
| **ADORA2A** | 11.88 | 2.12 | 1.29 | 0.85 |
| **C3AR1** | 11.86 | 0.73 | 0.76 | 3.75 |
| **LRRC32** | 11.85 | 1.43 | 0.82 | 1.32 |
| **MAP3K8** | 11.82 | 12.66 | 23.99 | 9.22 |
| **IRF1** | 11.71 | 13.70 | 14.50 | 8.78 |
| **MT1G** | 11.71 | 41.63 | 75.19 | 2.27 |
| **HERC6** | 11.06 | 0.73 | 1.07 | 1.04 |
| **CD274** | 10.92 | 5.30 | 4.25 | 6.73 |
| **NFKBIZ** | 10.76 | 22.87 | 34.25 | 8.34 |
| **RNF19B** | 10.56 | 3.70 | 3.43 | 2.37 |
| **PLSCR1** | 10.54 | 2.17 | 1.10 | 1.91 |
| **LOC389386** | 10.47 | 1.80 | 1.25 | 2.46 |
| **APOL3** | 10.39 | 1.36 | 0.90 | 1.76 |
| **LOC647086** | 10.35 | 6.95 | 7.97 | 6.40 |
| **BATF3** | 10.32 | 1.04 | 0.69 | 1.99 |
| **HLA-DMB** | 10.30 | 1.63 | 1.20 | 0.91 |
| **SLAMF8** | 10.27 | 0.15 | 0.44 | 0.60 |
| **KRT17** | 10.26 | 4.15 | 2.11 | 1.41 |
| **NUAK2** | 10.13 | 7.14 | 14.46 | 2.79 |
| **CD7** | 9.99 | 3.28 | 1.00 | 1.00 |
| **LOC650799** | 9.87 | 0.35 | 0.30 | 0.74 |
| **SPRY4** | 9.82 | 7.24 | 12.76 | 10.01 |
| **KIAA1618** | 9.76 | 1.62 | 0.55 | 2.83 |
| **ARL9** | 9.58 | 0.26 | 0.75 | 1.89 |
| **ADAP1** | 9.57 | 1.08 | 2.36 | 0.67 |
| **SERPINB2** | 9.57 | 0.62 | 0.58 | 2.47 |
| **ART3** | 9.55 | 0.85 | 0.58 | 0.85 |
| **SERPINE1** | 9.51 | 0.85 | 1.39 | 1.64 |
| **IFIT5** | 9.48 | 1.78 | 1.09 | 1.48 |
| **LIPG** | 9.39 | 0.22 | 0.51 | 0.56 |
| **PPAP2B** | 9.38 | 0.35 | 0.53 | 2.57 |
| **PARP14** | 9.35 | 1.19 | 0.89 | 1.49 |
| **MAFF** | 9.25 | 9.29 | 13.56 | 12.18 |
| **TYMP** | 9.00 | 0.42 | 0.72 | 1.59 |
| **TMEM140** | 8.99 | 5.04 | 1.04 | 2.89 |
| **LOC652671** | 8.92 | 1.67 | 1.30 | 1.45 |
| **MIR221** | 8.89 | 1.61 | 4.16 | 4.28 |
| **RASGRP3** | 8.88 | 0.32 | 0.32 | 0.32 |
| **JAK2** | 8.86 | 2.23 | 1.56 | 1.65 |
| **LOC732371** | 8.86 | 0.20 | 0.19 | 2.46 |
| **CLDN1** | 8.73 | 2.39 | 1.07 | 1.64 |
| **LOC374443** | 8.66 | 6.49 | 5.52 | 3.33 |
| **ESM1** | 8.61 | 3.22 | 7.16 | 5.58 |
| **LOC653778** | 8.61 | 1.01 | 0.27 | 0.94 |
| **ICOSLG** | 8.57 | 1.67 | 1.40 | 0.79 |
| **TRIB1** | 8.53 | 6.90 | 4.84 | 11.43 |
| **IL7R** | 8.44 | 0.07 | 0.25 | 1.01 |
| **KRT34** | 8.36 | 0.43 | 1.62 | 2.07 |
| **ISG15** | 8.32 | 1.83 | 2.92 | 0.96 |
| **TNC** | 8.29 | 0.50 | 0.64 | 0.77 |
| **SIX1** | 8.21 | 1.20 | 1.15 | 0.88 |
| **IRAK2** | 8.20 | 5.16 | 6.32 | 2.33 |
| **RIPK2** | 8.15 | 4.22 | 2.70 | 1.41 |
| **CCL3** | 8.09 | 0.69 | 2.67 | 0.81 |
| **LOC654103** | 7.81 | 1.13 | 0.41 | 1.03 |
| **DDX60** | 7.63 | 2.79 | 2.02 | 1.57 |
| **SECTM1** | 7.62 | 1.10 | 0.63 | 1.09 |
| **FAM107A** | 7.58 | 0.29 | 0.20 | 1.13 |
| **C6orf141** | 7.48 | 0.69 | 0.45 | 3.61 |
| **SAMHD1** | 7.46 | 0.63 | 0.37 | 0.94 |
| **C20orf141** | 7.42 | 1.89 | 0.52 | 2.63 |
| **SLC25A37** | 7.37 | 2.83 | 1.26 | 1.26 |
| **FOSL1** | 7.36 | 1.84 | 2.38 | 6.10 |
| **MT1M** | 7.35 | 6.21 | 23.76 | 1.15 |
| **KLF10** | 7.34 | 1.33 | 3.13 | 5.81 |
| **LOC441019** | 7.31 | 4.62 | 4.54 | 1.02 |
| **ZC3HAV1** | 7.28 | 1.70 | 2.94 | 2.11 |
| **LOC162073** | 7.26 | 6.10 | 3.93 | 5.95 |
| **BCL3** | 7.21 | 2.58 | 2.36 | 1.17 |
| **TBKBP1** | 7.17 | 2.30 | 3.65 | 3.61 |
| **PRIC285** | 7.16 | 3.73 | 2.35 | 1.83 |
| **CEACAM1** | 7.09 | 0.24 | 2.26 | 0.95 |
| **LAP3** | 6.95 | 1.85 | 1.09 | 2.45 |
| **STC1** | 6.93 | 0.25 | 2.96 | 1.64 |
| **TP63** | 6.88 | 0.37 | 0.61 | 0.54 |
| **PDE6H** | 6.87 | 4.02 | 3.54 | 1.79 |
| **GBP3** | 6.86 | 2.46 | 0.78 | 1.71 |
| **NKX3-1** | 6.79 | 2.13 | 2.06 | 1.41 |
| **KIAA1217** | 6.71 | 0.27 | 0.02 | 0.53 |
| **TAP1** | 6.69 | 4.02 | 2.61 | 2.41 |
| **CTRL** | 6.64 | 0.31 | 0.51 | 3.54 |
| **C14orf73** | 6.61 | 1.08 | 0.70 | 0.70 |
| **NLRP3** | 6.54 | 0.86 | 1.36 | 1.08 |
| **EDNRA** | 6.53 | 1.17 | 0.78 | 0.82 |
| **ZNFX1** | 6.53 | 1.61 | 2.09 | 1.49 |
| **C13orf16** | 6.45 | 0.41 | 0.57 | 1.05 |
| **LAMP3** | 6.43 | 4.81 | 8.02 | 0.98 |
| **S1PR1** | 6.40 | 1.17 | 0.75 | 2.09 |
| **OAS3** | 6.36 | 1.28 | 0.74 | 0.84 |
| **NFKB2** | 6.35 | 3.33 | 3.28 | 1.46 |
| **FGF2** | 6.34 | 1.08 | 0.84 | 1.98 |
| **MIR149** | 6.29 | 2.37 | 1.52 | 1.54 |
| **MSX1** | 6.20 | 1.92 | 5.25 | 2.87 |
| **SAMD9** | 6.16 | 0.67 | 0.59 | 0.73 |
| **AXUD1** | 6.11 | 2.84 | 8.33 | 5.92 |
| **STAT1** | 6.10 | 4.04 | 1.53 | 2.22 |
| **TRIM16** | 6.06 | 0.86 | 1.67 | 2.14 |
| **PARP9** | 6.05 | 2.92 | 0.96 | 2.43 |
| **SDC4** | 5.93 | 3.52 | 2.45 | 1.57 |
| **DDX60L** | 5.87 | 0.75 | 0.25 | 0.82 |
| **MLKL** | 5.84 | 0.46 | 0.22 | 0.97 |
| **VEGFC** | 5.83 | 0.47 | 0.55 | 1.57 |
| **FAM150B** | 5.81 | 1.31 | 1.31 | 1.32 |
| **PLAUR** | 5.80 | 0.94 | 1.44 | 1.81 |
| **NEDD9** | 5.79 | 4.16 | 5.23 | 1.89 |
| **GFPT2** | 5.78 | 2.66 | 2.01 | 1.77 |
| **CENTA1** | 5.77 | 0.97 | 2.18 | 1.14 |
| **CCRN4L** | 5.76 | 3.77 | 6.02 | 3.98 |
| **ZMYND15** | 5.73 | 6.15 | 4.22 | 2.89 |
| **APOBEC3G** | 5.73 | 1.00 | 1.08 | 1.37 |
| **LOC100133591** | 5.70 | 0.98 | 0.73 | 2.54 |
| **PMAIP1** | 5.68 | 4.53 | 6.20 | 6.41 |
| **FLJ32255** | 5.65 | 2.35 | 0.71 | 1.78 |
| **SPSB1** | 5.63 | 1.49 | 1.75 | 3.27 |
| **FAS** | 5.54 | 0.82 | 2.02 | 0.78 |
| **TNIP1** | 5.54 | 1.97 | 2.84 | 1.06 |
| **TGM2** | 5.50 | 1.05 | 0.68 | 2.74 |
| **ELF3** | 5.50 | 1.37 | 0.32 | 0.78 |
| **PML** | 5.46 | 1.24 | 1.10 | 1.09 |
| **TRIM25** | 5.45 | 1.97 | 1.19 | 1.25 |
| **EPSTI1** | 5.44 | 1.12 | 0.53 | 1.53 |
| **DTX3L** | 5.43 | 2.10 | 2.19 | 1.87 |
| **HLA-DRA** | 5.42 | 0.98 | 1.07 | 1.10 |
| **IRF7** | 5.39 | 2.35 | 3.32 | 1.32 |
| **PLEKHF1** | 5.34 | 2.75 | 2.08 | 2.77 |
| **DENND3** | 5.28 | 2.32 | 1.02 | 1.15 |
| **NLRC5** | 5.21 | 3.33 | 0.81 | 0.80 |
| **SBNO2** | 5.19 | 3.53 | 1.75 | 2.68 |
| **IFI35** | 5.13 | 1.32 | 0.49 | 1.61 |
| **SLC6A2** | 5.13 | 2.09 | 6.68 | 2.96 |
| **DRAM1** | 5.12 | 1.44 | 1.65 | 1.12 |
| **IL15** | 5.08 | 2.19 | 1.32 | 1.70 |
| **RHBDF2** | 5.04 | 1.40 | 1.21 | 1.17 |
| **SLC15A3** | 5.01 | 0.93 | 0.88 | 1.23 |
| **STAT5A** | 5.00 | 2.28 | 1.96 | 0.97 |
| **PTGER4** | 4.99 | 2.00 | 3.80 | 2.37 |
| **RELB** | 4.96 | 6.04 | 5.54 | 1.64 |
| **NAMPT** | 4.95 | 1.80 | 3.00 | 1.70 |
| **CEBPD** | 4.95 | 3.21 | 1.89 | 1.41 |
| **SP110** | 4.94 | 1.49 | 0.92 | 1.57 |
| **FGF5** | 4.94 | 0.62 | 1.20 | 1.44 |
| **SOCS3** | 4.93 | 1.93 | 1.35 | 5.16 |
| **TRIM21** | 4.89 | 2.58 | 2.06 | 3.11 |
| **TAP2** | 4.86 | 1.70 | 0.95 | 1.30 |
| **SLC25A28** | 4.84 | 1.55 | 1.00 | 1.38 |
| **RRAD** | 4.81 | 2.21 | 4.17 | 7.96 |
| **ZC3H12C** | 4.80 | 2.46 | 2.49 | 1.60 |
| **KIAA1199** | 4.78 | 0.06 | 0.11 | 0.48 |
| **ASPHD2** | 4.78 | 1.26 | 0.80 | 1.13 |
| **OXTR** | 4.74 | 0.78 | 0.83 | 1.02 |
| **LOC729941** | 4.72 | 1.55 | 1.21 | 4.52 |
| **LOC100127894** | 4.70 | 1.48 | 1.63 | 3.47 |
| **CFLAR** | 4.69 | 1.46 | 0.90 | 1.14 |
| **SLC25A24** | 4.69 | 2.60 | 2.72 | 1.15 |
| **PANX1** | 4.66 | 1.69 | 1.97 | 1.60 |
| **SAMD8** | 4.64 | 5.71 | 6.44 | 5.61 |
| **LOC100134000** | 4.63 | 3.26 | 2.73 | 0.33 |
| **SSTR2** | 4.62 | 1.68 | 0.84 | 1.05 |
| **P2RX7** | 4.56 | 1.06 | 0.29 | 1.30 |
| **RNU86** | 4.54 | 7.29 | 23.69 | 3.78 |
| **TMEM16D** | 4.48 | 0.34 | 0.88 | 1.04 |
| **ECGF1** | 4.46 | 0.56 | 0.68 | 1.10 |
| **SAMD4A** | 4.42 | 1.70 | 1.00 | 1.60 |
| **MTE** | 4.41 | 5.47 | 9.24 | 0.85 |
| **TMEM217** | 4.41 | 0.34 | 0.31 | 1.09 |
| **NFKBIA** | 4.38 | 4.55 | 5.20 | 1.07 |
| **LOC100129303** | 4.37 | 1.30 | 1.66 | 2.44 |
| **RNF213** | 4.36 | 0.95 | 0.44 | 1.29 |
| **TIFA** | 4.27 | 3.50 | 3.11 | 0.47 |
| **LOC643977** | 4.25 | 1.94 | 0.52 | 1.61 |
| **ADPRH** | 4.25 | 0.87 | 0.58 | 1.29 |
| **EDG1** | 4.23 | 1.07 | 0.91 | 1.45 |
| **HOXD10** | 4.23 | 1.19 | 3.40 | 0.57 |
| **PARP12** | 4.22 | 1.46 | 0.83 | 1.15 |
| **ADRB2** | 4.22 | 1.40 | 0.90 | 1.10 |
| **TMEM158** | 4.22 | 0.63 | 1.68 | 1.07 |
| **CD83** | 4.20 | 6.10 | 9.61 | 1.62 |
| **FUT4** | 4.17 | 1.00 | 1.63 | 0.90 |
| **OGFR** | 4.17 | 1.87 | 1.37 | 1.25 |
| **DUSP5** | 4.16 | 2.99 | 3.08 | 4.54 |
| **MAFB** | 4.15 | 0.65 | 1.36 | 6.02 |
| **DUSP19** | 4.15 | 1.76 | 1.27 | 1.26 |
| **STOML1** | 4.15 | 2.01 | 0.76 | 0.87 |
| **PLEKHA4** | 4.14 | 1.32 | 0.90 | 1.20 |
| **ABTB2** | 4.13 | 1.17 | 1.93 | 1.43 |
| **DUSP6** | 4.12 | 2.67 | 2.16 | 4.44 |
| **IRF2** | 4.10 | 1.39 | 1.03 | 1.00 |
| **SH3BP2** | 4.08 | 1.25 | 1.08 | 1.25 |
| **LOC730256** | 4.08 | 4.19 | 2.29 | 1.84 |
| **CITED4** | 4.04 | 0.83 | 0.63 | 0.85 |
| **C15orf39** | 3.97 | 2.79 | 3.29 | 1.82 |
| **EDN1** | 3.97 | 0.81 | 2.55 | 1.25 |
| **GUCY1A2** | 3.97 | 0.66 | 1.46 | 1.11 |
| **TRIM14** | 3.96 | 0.80 | 0.61 | 1.04 |
| **CA13** | 3.95 | 1.22 | 1.12 | 0.94 |
| **NCRNA00152** | 3.94 | 0.56 | 0.85 | 1.18 |
| **SP100** | 3.93 | 0.69 | 0.52 | 0.80 |
| **TRIM56** | 3.93 | 1.84 | 1.11 | 1.31 |
| **S1PR3** | 3.92 | 1.25 | 1.69 | 1.46 |
| **LAYN** | 3.92 | 1.18 | 0.58 | 1.61 |
| **FAM176A** | 3.91 | 0.57 | 0.66 | 1.45 |
| **RILP** | 3.89 | 1.50 | 1.12 | 1.46 |
| **SPHK1** | 3.89 | 1.01 | 2.06 | 3.05 |
| **IL12A** | 3.88 | 3.84 | 6.08 | 3.67 |
| **PAPPA** | 3.84 | 0.18 | 0.53 | 0.95 |
| **SLC43A3** | 3.82 | 1.03 | 1.46 | 0.97 |
| **TIMP3** | 3.80 | 1.51 | 0.26 | 0.83 |
| **NFKB1** | 3.80 | 1.99 | 2.68 | 1.01 |
| **SLFN5** | 3.78 | 2.63 | 3.10 | 1.08 |
| **GIMAP2** | 3.74 | 1.18 | 0.88 | 2.36 |
| **LOC729692** | 3.74 | 1.52 | 1.75 | 1.76 |
| **NGF** | 3.73 | 2.28 | 2.83 | 1.48 |
| **IL24** | 3.73 | 0.31 | 0.28 | 1.07 |
| **C19orf66** | 3.72 | 1.14 | 0.94 | 1.37 |
| **RELA** | 3.71 | 1.89 | 2.29 | 1.46 |
| **C21orf91** | 3.69 | 1.03 | 1.75 | 1.22 |
| **PSMB9** | 3.68 | 2.46 | 1.01 | 1.50 |
| **NINJ1** | 3.67 | 2.11 | 2.59 | 0.86 |
| **NCOA7** | 3.66 | 1.46 | 1.54 | 1.26 |
| **EBI3** | 3.66 | 0.91 | 1.04 | 0.75 |
| **SMURF2** | 3.66 | 0.88 | 0.73 | 1.82 |
| **C5orf39** | 3.65 | 1.21 | 0.63 | 1.03 |
| **PHLDA1** | 3.64 | 1.62 | 1.64 | 1.60 |
| **SOX7** | 3.64 | 1.79 | 2.63 | 1.72 |
| **TLR4** | 3.64 | 0.96 | 1.06 | 1.85 |
| **LOC100128055** | 3.64 | 0.44 | 0.33 | 0.52 |
| **MYD88** | 3.63 | 0.70 | 0.49 | 1.04 |
| **ST3GAL1** | 3.63 | 2.34 | 1.96 | 2.06 |
| **TRAFD1** | 3.61 | 1.68 | 1.40 | 1.51 |
| **SPOCD1** | 3.56 | 0.55 | 1.82 | 1.22 |
| **UBE2L6** | 3.56 | 1.98 | 1.10 | 1.70 |
| **TRIM38** | 3.55 | 1.32 | 0.73 | 1.06 |
| **FLJ35409** | 3.54 | 0.60 | 0.11 | 0.67 |
| **HLA-DMA** | 3.54 | 0.61 | 0.79 | 0.99 |
| **LOX** | 3.53 | 0.57 | 3.33 | 1.09 |
| **NFKBIE** | 3.51 | 2.10 | 3.07 | 1.16 |
| **LOC645574** | 3.50 | 2.65 | 1.23 | 2.15 |
| **MMAA** | 3.50 | 3.35 | 2.61 | 2.39 |
| **SHB** | 3.48 | 1.22 | 1.47 | 1.15 |
| **CD47** | 3.48 | 1.22 | 0.41 | 0.97 |
| **ZNF600** | 3.47 | 1.61 | 2.99 | 0.83 |
| **IFI16** | 3.46 | 1.38 | 1.31 | 1.59 |
| **CDCP1** | 3.43 | 1.54 | 1.48 | 1.21 |
| **NMI** | 3.42 | 1.66 | 1.36 | 1.47 |
| **NAV3** | 3.42 | 0.27 | 0.11 | 3.58 |
| **AEN** | 3.42 | 1.41 | 3.50 | 1.63 |
| **CD70** | 3.41 | 1.35 | 1.26 | 1.01 |
| **CXCR7** | 3.40 | 0.37 | 1.62 | 0.94 |
| **STAT2** | 3.39 | 1.83 | 0.93 | 1.27 |
| **RNF122** | 3.39 | 2.09 | 5.16 | 1.94 |
| **SRFBP1** | 3.39 | 1.53 | 1.86 | 1.20 |
| **TSKU** | 3.35 | 1.06 | 1.42 | 1.17 |
| **SLC2A6** | 3.35 | 0.60 | 0.62 | 0.96 |
| **PDZD2** | 3.33 | 0.39 | 0.22 | 0.51 |
| **PPM2C** | 3.33 | 1.01 | 0.91 | 1.69 |
| **IKBKE** | 3.32 | 0.58 | 0.46 | 0.76 |
| **LOC650832** | 3.31 | 1.40 | 1.09 | 2.44 |
| **LOC729196** | 3.27 | 1.78 | 1.34 | 1.09 |
| **CXorf38** | 3.27 | 2.19 | 1.32 | 1.03 |
| **SOX17** | 3.26 | 0.86 | 0.28 | 0.75 |
| **C6orf132** | 3.24 | 0.46 | 1.61 | 1.10 |
| **LOC100128918** | 3.24 | 1.07 | 0.97 | 1.35 |
| **P2RY5** | 3.24 | 0.99 | 0.30 | 0.80 |
| **DENND2D** | 3.23 | 1.28 | 1.13 | 0.99 |
| **NFKBID** | 3.21 | 4.31 | 7.80 | 1.62 |
| **B4GALT5** | 3.21 | 1.11 | 0.83 | 1.42 |
| **ZNF697** | 3.21 | 3.52 | 4.82 | 2.36 |
| **LOC650254** | 3.20 | 0.98 | 1.60 | 1.31 |
| **MGC16291** | 3.17 | 0.11 | 0.11 | 0.89 |
| **SQRDL** | 3.16 | 1.30 | 0.62 | 1.21 |
| **KIAA0247** | 3.15 | 2.24 | 2.54 | 1.29 |
| **PLK3** | 3.14 | 4.93 | 2.41 | 2.99 |
| **F3** | 3.12 | 2.08 | 2.56 | 1.99 |
| **BIRC2** | 3.11 | 1.86 | 1.51 | 1.12 |
| **C19orf12** | 3.11 | 0.95 | 1.14 | 1.26 |
| **LBA1** | 3.11 | 1.01 | 1.15 | 1.02 |
| **ADAMTS6** | 3.10 | 0.49 | 0.60 | 1.56 |
| **PVT1** | 3.09 | 1.29 | 3.74 | 2.24 |
| **GMPR** | 3.09 | 0.87 | 0.55 | 1.04 |
| **S100A3** | 3.09 | 0.10 | 0.12 | 0.94 |
| **GRB10** | 3.08 | 1.21 | 0.98 | 0.75 |
| **LOC154761** | 3.08 | 1.25 | 3.32 | 2.60 |
| **LOC100131091** | 3.08 | 1.31 | 0.84 | 1.17 |
| **ZNF296** | 3.06 | 0.99 | 2.50 | 3.52 |
| **MT1A** | 3.06 | 2.70 | 2.98 | 0.95 |
| **TDRD7** | 3.06 | 1.33 | 1.23 | 0.92 |
| **JUNB** | 3.05 | 3.27 | 2.88 | 2.40 |
| **SERPINB9** | 3.05 | 0.96 | 0.37 | 1.51 |
| **HIVEP1** | 3.05 | 1.49 | 1.25 | 0.86 |
| **CPEB2** | 3.03 | 1.39 | 2.39 | 2.90 |
| **PDGFB** | 3.03 | 0.59 | 1.11 | 0.83 |
| **NFATC1** | 3.01 | 1.58 | 1.04 | 0.77 |
| **RARRES1** | 3.01 | 0.60 | 0.41 | 0.91 |
| **ADC** | 3.01 | 1.78 | 1.32 | 0.79 |
| **ETS1** | 3.01 | 2.16 | 3.21 | 1.84 |
| **RHEBL1** | 3.00 | 3.13 | 1.78 | 1.36 |
| **HLA-E** | 3.00 | 2.47 | 1.90 | 2.07 |
| **FAM139A** | 3.00 | 0.76 | 2.88 | 1.98 |
| **PIM1** | 2.99 | 6.14 | 5.83 | 5.94 |
| **PDLIM5** | 2.99 | 1.36 | 2.52 | 3.50 |
| **LRRC3** | 2.98 | 0.31 | 0.84 | 0.80 |
| **EFNA1** | 2.97 | 3.49 | 4.16 | 0.92 |
| **C20orf127** | 2.97 | 4.32 | 8.69 | 0.88 |
| **PARP10** | 2.96 | 1.09 | 1.19 | 0.91 |
| **MAP2K3** | 2.96 | 1.22 | 0.99 | 1.99 |
| **PTPRE** | 2.95 | 0.46 | 0.40 | 0.82 |
| **PSMB8** | 2.95 | 1.35 | 0.71 | 1.31 |
| **C14orf4** | 2.95 | 1.34 | 0.77 | 0.59 |
| **RNF114** | 2.94 | 1.19 | 1.07 | 1.43 |
| **LOC100131330** | 2.94 | 0.49 | 1.22 | 0.60 |
| **SMAD3** | 2.93 | 0.63 | 0.64 | 0.76 |
| **FHL3** | 2.92 | 1.09 | 1.12 | 1.87 |
| **MAML2** | 2.90 | 0.36 | 0.55 | 0.41 |
| **TMEM166** | 2.89 | 0.61 | 0.63 | 1.19 |
| **ERAP2** | 2.88 | 0.96 | 0.67 | 1.08 |
| **MAP3K5** | 2.88 | 0.73 | 0.58 | 1.05 |
| **PTPRH** | 2.87 | 2.42 | 3.24 | 1.57 |
| **IFI6** | 2.87 | 0.86 | 0.75 | 1.09 |
| **FOSL2** | 2.86 | 1.35 | 2.02 | 1.98 |
| **LONRF1** | 2.85 | 2.67 | 2.52 | 2.08 |
| **PLAT** | 2.85 | 0.93 | 1.07 | 1.17 |
| **PHF15** | 2.85 | 0.45 | 0.12 | 0.51 |
| **LGALS8** | 2.84 | 1.52 | 2.95 | 1.37 |
| **TMEM62** | 2.83 | 1.29 | 1.38 | 1.01 |
| **B3GNT2** | 2.83 | 0.82 | 1.52 | 1.42 |
| **SLC37A1** | 2.83 | 1.48 | 1.99 | 1.18 |
| **DNPEP** | 2.82 | 1.51 | 1.82 | 1.23 |
| **LOC100134728** | 2.80 | 0.62 | 0.62 | 1.37 |
| **GOLM1** | 2.79 | 1.16 | 0.92 | 1.01 |
| **MT2A** | 2.79 | 2.50 | 2.63 | 0.89 |
| **MT1X** | 2.79 | 11.15 | 14.46 | 1.22 |
| **SLC39A14** | 2.78 | 1.59 | 1.42 | 1.25 |
| **SLC22A4** | 2.76 | 0.60 | 0.32 | 0.89 |
| **ST5** | 2.76 | 0.75 | 0.40 | 0.99 |
| **CASP4** | 2.76 | 1.52 | 1.32 | 1.60 |
| **LOC644629** | 2.75 | 2.08 | 2.68 | 2.62 |
| **TGIF1** | 2.74 | 1.49 | 0.98 | 1.18 |
| **SYTL3** | 2.74 | 0.95 | 1.27 | 1.13 |
| **FLJ35258** | 2.74 | 0.42 | 0.52 | 0.96 |
| **LOC100132394** | 2.74 | 4.28 | 2.05 | 5.65 |
| **C1orf222** | 2.71 | 1.64 | 1.35 | 1.76 |
| **LOC646626** | 2.70 | 0.29 | 0.11 | 1.08 |
| **ETS2** | 2.70 | 1.01 | 1.09 | 1.59 |
| **ELL** | 2.70 | 1.10 | 1.61 | 2.53 |
| **SLC41A2** | 2.69 | 1.45 | 1.20 | 0.93 |
| **RNY4** | 2.69 | 9.22 | 2.28 | 15.26 |
| **ITPRIPL2** | 2.68 | 1.32 | 1.25 | 1.45 |
| **KLF6** | 2.68 | 2.25 | 2.31 | 2.37 |
| **SAT1** | 2.68 | 2.31 | 2.06 | 1.92 |
| **CCNL1** | 2.67 | 2.23 | 2.81 | 3.63 |
| **SEC14L2** | 2.67 | 0.61 | 1.37 | 1.19 |
| **BAK1** | 2.67 | 1.08 | 1.07 | 1.20 |
| **HOXD11** | 2.67 | 0.68 | 3.15 | 0.53 |
| **LOC100132663** | 2.66 | 2.74 | 3.61 | 2.58 |
| **UNC93B1** | 2.66 | 0.87 | 1.16 | 1.02 |
| **LOC400879** | 2.66 | 0.80 | 0.78 | 1.09 |
| **BCAR3** | 2.65 | 1.48 | 2.74 | 2.67 |
| **SLC31A2** | 2.64 | 1.19 | 1.56 | 1.12 |
| **LAMC2** | 2.64 | 1.02 | 1.24 | 0.84 |
| **ETV6** | 2.63 | 0.99 | 0.93 | 1.29 |
| **LOC645284** | 2.62 | 1.04 | 1.53 | 2.19 |
| **THSD1** | 2.62 | 1.05 | 0.83 | 0.95 |
| **ZEB2** | 2.62 | 0.91 | 1.15 | 1.05 |
| **USP15** | 2.61 | 1.08 | 1.12 | 1.20 |
| **PHLDB2** | 2.61 | 0.79 | 0.50 | 0.84 |
| **ZSWIM4** | 2.61 | 1.45 | 1.23 | 1.90 |
| **SMOX** | 2.61 | 1.66 | 5.10 | 2.94 |
| **NRP2** | 2.60 | 0.91 | 0.77 | 1.34 |
| **PSTPIP2** | 2.59 | 1.33 | 1.81 | 0.86 |
| **ARHGAP28** | 2.59 | 0.70 | 0.28 | 0.84 |
| **SLC11A2** | 2.58 | 2.00 | 2.58 | 1.38 |
| **CASP7** | 2.58 | 1.11 | 1.00 | 1.62 |
| **FJX1** | 2.57 | 0.41 | 0.61 | 0.49 |
| **GAL** | 2.57 | 0.25 | 0.86 | 1.23 |
| **VEGFA** | 2.56 | 3.66 | 16.98 | 6.22 |
| **CBR3** | 2.56 | 0.63 | 0.61 | 1.18 |
| **OPTN** | 2.55 | 1.38 | 0.96 | 1.02 |
| **CDC42SE2** | 2.55 | 1.38 | 0.96 | 1.71 |
| **NNMT** | 2.54 | 0.75 | 0.71 | 1.57 |
| **WTAP** | 2.54 | 1.10 | 1.31 | 1.17 |
| **TMEM233** | 2.54 | 0.58 | 1.25 | 0.96 |
| **NPPB** | 2.52 | 0.15 | 2.44 | 0.73 |
| **LOC643206** | 2.52 | 3.04 | 3.95 | 2.85 |
| **C3orf52** | 2.51 | 1.00 | 1.00 | 2.30 |
| **KLF9** | 2.50 | 1.56 | 1.05 | 0.85 |
| **TNFRSF14** | 2.50 | 0.63 | 1.59 | 1.14 |
| **KCTD14** | 2.50 | 0.38 | 0.38 | 0.69 |
| **CCND1** | 2.49 | 0.62 | 0.38 | 0.81 |
| **LOC643384** | 2.49 | 0.93 | 1.12 | 0.95 |
| **B3GALNT1** | 2.49 | 0.73 | 0.61 | 0.81 |
| **IER3** | 2.48 | 3.18 | 2.73 | 1.46 |
| **BCL10** | 2.48 | 0.85 | 1.31 | 2.13 |
| **POLR3D** | 2.47 | 1.42 | 1.43 | 1.71 |
| **NFIL3** | 2.47 | 3.27 | 6.10 | 3.07 |
| **RAB38** | 2.47 | 1.65 | 1.64 | 0.86 |
| **MIDN** | 2.47 | 1.18 | 1.13 | 2.58 |
| **FLJ90086** | 2.46 | 1.17 | 0.98 | 1.01 |
| **NUB1** | 2.46 | 1.81 | 0.99 | 1.16 |
| **NR3C1** | 2.46 | 0.90 | 1.10 | 1.02 |
| **ARHGEF11** | 2.46 | 0.93 | 0.99 | 0.69 |
| **TICAM1** | 2.46 | 1.68 | 2.73 | 2.01 |
| **CTTNBP2NL** | 2.46 | 0.90 | 1.04 | 1.17 |
| **OSMR** | 2.45 | 1.06 | 2.57 | 1.29 |
| **SLC35E4** | 2.44 | 1.09 | 1.04 | 1.76 |
| **ITPRIP** | 2.44 | 2.24 | 3.28 | 2.35 |
| **DCP1A** | 2.44 | 1.73 | 1.80 | 1.51 |
| **EPHA2** | 2.42 | 2.30 | 1.86 | 2.09 |
| **THBD** | 2.42 | 0.47 | 0.28 | 1.13 |
| **RND3** | 2.42 | 2.60 | 4.45 | 5.77 |
| **SEPT4** | 2.42 | 1.51 | 1.01 | 1.19 |
| **PIK3CD** | 2.42 | 1.40 | 1.54 | 1.17 |
| **HRH1** | 2.42 | 0.98 | 1.25 | 1.89 |
| **OBFC2A** | 2.42 | 0.68 | 0.38 | 1.81 |
| **IRF2BP2** | 2.42 | 1.52 | 1.11 | 0.72 |
| **FAM65B** | 2.42 | 0.72 | 0.27 | 0.60 |
| **RAD9A** | 2.42 | 1.50 | 1.23 | 1.67 |
| **GRAMD3** | 2.41 | 0.73 | 0.82 | 2.01 |
| **KIAA1609** | 2.40 | 1.39 | 1.27 | 0.91 |
| **AJAP1** | 2.40 | 1.09 | 0.96 | 1.22 |
| **LYSMD2** | 2.40 | 0.68 | 0.58 | 0.92 |
| **SNORA77** | 2.40 | 1.51 | 0.97 | 0.24 |
| **KLF15** | 2.40 | 3.37 | 3.94 | 0.93 |
| **TGFB2** | 2.40 | 0.30 | 0.50 | 0.82 |
| **TMEM51** | 2.40 | 0.93 | 0.78 | 1.43 |
| **TRIM5** | 2.39 | 0.66 | 0.58 | 1.02 |
| **LGALS3BP** | 2.39 | 1.03 | 0.92 | 1.24 |
| **TRIM26** | 2.39 | 1.46 | 1.75 | 1.50 |
| **C16orf7** | 2.39 | 1.67 | 1.16 | 1.36 |
| **LOC441007** | 2.39 | 1.31 | 1.55 | 0.85 |
| **TNFRSF6B** | 2.38 | 1.39 | 1.05 | 1.06 |
| **HIF1A** | 2.38 | 1.01 | 0.77 | 1.49 |
| **DCUN1D3** | 2.38 | 1.38 | 1.77 | 2.48 |
| **LOC642477** | 2.38 | 0.75 | 0.48 | 1.16 |
| **RCAN1** | 2.37 | 0.64 | 1.10 | 1.01 |
| **LOC653496** | 2.37 | 1.75 | 1.48 | 1.24 |
| **XBP1** | 2.37 | 2.63 | 3.23 | 2.23 |
| **MCL1** | 2.37 | 1.68 | 1.94 | 2.57 |
| **TRIP10** | 2.36 | 1.55 | 1.18 | 1.50 |
| **CDGAP** | 2.36 | 0.64 | 0.39 | 0.73 |
| **CARD16** | 2.36 | 0.72 | 0.47 | 1.59 |
| **PHLDA2** | 2.35 | 0.52 | 0.53 | 2.02 |
| **C1RL** | 2.35 | 1.04 | 0.56 | 1.00 |
| **FAM113A** | 2.35 | 1.54 | 0.96 | 1.18 |
| **LACTB** | 2.35 | 1.58 | 1.19 | 1.06 |
| **BTN3A3** | 2.35 | 1.04 | 0.85 | 0.89 |
| **LMBR1L** | 2.34 | 1.24 | 1.19 | 1.27 |
| **ZFP36** | 2.34 | 4.20 | 6.46 | 5.72 |
| **RNF24** | 2.34 | 1.08 | 3.14 | 1.15 |
| **C19orf28** | 2.34 | 1.07 | 0.76 | 1.21 |
| **STBD1** | 2.34 | 1.16 | 3.30 | 0.96 |
| **GREM1** | 2.34 | 0.88 | 0.96 | 2.17 |
| **SERPINB8** | 2.34 | 0.40 | 1.46 | 1.80 |
| **MGC39372** | 2.34 | 0.34 | 0.17 | 0.70 |
| **ELF4** | 2.34 | 1.17 | 1.56 | 1.40 |
| **TNFRSF1B** | 2.34 | 1.21 | 0.81 | 1.15 |
| **BAZ1A** | 2.33 | 0.93 | 0.77 | 1.37 |
| **CDC42EP2** | 2.33 | 0.51 | 0.39 | 1.04 |
| **SLC25A22** | 2.33 | 1.10 | 1.14 | 1.44 |
| **IER2** | 2.33 | 1.46 | 1.71 | 1.53 |
| **LOC100132565** | 2.33 | 1.35 | 1.41 | 1.54 |
| **ADM** | 2.33 | 1.96 | 2.95 | 4.87 |
| **NOLC1** | 2.33 | 1.11 | 1.26 | 1.25 |
| **HIVEP2** | 2.32 | 1.42 | 1.37 | 0.72 |
| **AUTS2** | 2.31 | 0.45 | 0.58 | 0.91 |
| **C14orf149** | 2.31 | 0.30 | 0.15 | 0.93 |
| **ABI3** | 2.31 | 0.58 | 0.45 | 0.64 |
| **NOD1** | 2.30 | 1.02 | 0.89 | 1.12 |
| **PKDCC** | 2.30 | 1.20 | 1.10 | 1.33 |
| **NR2F1** | 2.30 | 0.35 | 0.26 | 0.81 |
| **IFFO2** | 2.29 | 2.57 | 3.28 | 1.72 |
| **CEBPB** | 2.29 | 3.98 | 3.83 | 2.66 |
| **CD276** | 2.29 | 1.94 | 0.94 | 1.26 |
| **C4orf32** | 2.28 | 1.02 | 1.02 | 1.00 |
| **RAB43** | 2.28 | 1.84 | 1.74 | 1.58 |
| **SFT2D2** | 2.28 | 1.09 | 0.88 | 0.99 |
| **C11orf17** | 2.28 | 1.12 | 0.69 | 1.00 |
| **TXNIP** | 2.28 | 0.81 | 2.15 | 0.94 |
| **SH3TC1** | 2.27 | 5.67 | 5.68 | 1.88 |
| **LOC401317** | 2.27 | 3.11 | 3.85 | 1.52 |
| **CTGLF7** | 2.27 | 0.86 | 0.84 | 0.95 |
| **NAB1** | 2.27 | 1.74 | 1.26 | 1.56 |
| **CLDN23** | 2.26 | 1.99 | 0.81 | 0.71 |
| **LOC91461** | 2.25 | 0.80 | 1.27 | 1.42 |
| **EHD4** | 2.24 | 1.09 | 0.95 | 1.07 |
| **C4A** | 2.23 | 1.87 | 3.02 | 1.95 |
| **TRIM47** | 2.22 | 1.04 | 0.43 | 0.80 |
| **CAMK2D** | 2.22 | 1.40 | 1.14 | 2.04 |
| **STARD5** | 2.22 | 0.54 | 0.31 | 1.18 |
| **XRN1** | 2.22 | 1.24 | 1.17 | 0.90 |
| **SLFN12** | 2.21 | 0.49 | 0.32 | 1.49 |
| **SP140L** | 2.21 | 0.94 | 0.90 | 0.92 |
| **MAP2** | 2.21 | 2.27 | 1.48 | 2.36 |
| **MUC1** | 2.21 | 0.43 | 1.21 | 1.07 |
| **ISG20L1** | 2.20 | 1.09 | 2.56 | 1.31 |
| **CYP27B1** | 2.20 | 0.26 | 0.88 | 0.82 |
| **THBS1** | 2.20 | 0.41 | 0.57 | 1.15 |
| **IRF9** | 2.20 | 2.11 | 2.20 | 1.75 |
| **GPRC5B** | 2.20 | 1.52 | 0.73 | 1.45 |
| **LOC100129907** | 2.20 | 1.57 | 1.85 | 3.88 |
| **RNF149** | 2.19 | 1.65 | 1.25 | 1.42 |
| **TGFA** | 2.19 | 1.87 | 1.81 | 1.27 |
| **DCBLD1** | 2.18 | 1.60 | 0.84 | 1.42 |
| **RNU1A3** | 2.18 | 4.87 | 2.09 | 2.69 |
| **ALPK2** | 2.18 | 0.88 | 0.72 | 0.79 |
| **PSMB10** | 2.18 | 1.07 | 0.83 | 1.21 |
| **P2RX4** | 2.18 | 2.35 | 1.25 | 1.33 |
| **GABPB1** | 2.18 | 1.83 | 2.71 | 1.72 |
| **PSMA2** | 2.17 | 0.42 | 0.40 | 0.94 |
| **RNF144B** | 2.17 | 0.92 | 1.02 | 3.13 |
| **KLF3** | 2.17 | 1.68 | 1.15 | 1.81 |
| **GNB4** | 2.17 | 0.89 | 0.73 | 0.92 |
| **ZNF655** | 2.17 | 1.25 | 0.84 | 1.09 |
| **LOC100132738** | 2.17 | 0.91 | 1.05 | 0.79 |
| **C3orf38** | 2.17 | 1.02 | 1.40 | 0.82 |
| **CD82** | 2.17 | 0.70 | 0.86 | 0.99 |
| **PIM2** | 2.17 | 0.95 | 1.24 | 1.47 |
| **C1QTNF6** | 2.17 | 0.31 | 0.12 | 0.88 |
| **ZNF669** | 2.16 | 1.50 | 1.22 | 1.65 |
| **LOC201175** | 2.15 | 0.86 | 2.13 | 2.58 |
| **NEDD4L** | 2.15 | 0.69 | 0.39 | 0.80 |
| **RSC1A1** | 2.15 | 2.18 | 1.93 | 1.40 |
| **ZFP36L1** | 2.15 | 1.20 | 0.86 | 1.52 |
| **CD68** | 2.15 | 1.84 | 1.22 | 1.09 |
| **GPRC5A** | 2.15 | 0.47 | 0.75 | 1.47 |
| **SH2B3** | 2.14 | 1.58 | 1.20 | 1.69 |
| **LOC100134364** | 2.14 | 2.98 | 1.47 | 4.94 |
| **RBCK1** | 2.14 | 1.83 | 1.57 | 1.30 |
| **FOXP1** | 2.14 | 0.90 | 1.07 | 1.14 |
| **LIMK2** | 2.13 | 1.71 | 2.40 | 1.44 |
| **NOP16** | 2.13 | 0.74 | 0.74 | 1.05 |
| **C16orf55** | 2.13 | 1.40 | 1.79 | 1.11 |
| **CDKL1** | 2.12 | 0.45 | 0.58 | 0.94 |
| **ATP2B1** | 2.12 | 1.09 | 1.23 | 0.70 |
| **C3orf59** | 2.12 | 1.06 | 1.37 | 0.90 |
| **LOC440498** | 2.12 | 1.72 | 1.86 | 1.80 |
| **SCO2** | 2.12 | 0.74 | 0.51 | 0.69 |
| **NAV2** | 2.11 | 0.94 | 0.85 | 1.07 |
| **LITAF** | 2.11 | 1.21 | 0.83 | 1.44 |
| **WDR43** | 2.11 | 1.04 | 1.67 | 1.11 |
| **DAXX** | 2.11 | 1.09 | 1.15 | 0.94 |
| **VRK2** | 2.11 | 2.38 | 2.25 | 1.30 |
| **ACSL3** | 2.11 | 0.88 | 0.95 | 1.39 |
| **TLK2** | 2.10 | 1.80 | 2.31 | 1.31 |
| **HOXD4** | 2.10 | 1.24 | 1.59 | 1.14 |
| **B4GALT1** | 2.10 | 1.43 | 0.74 | 0.92 |
| **BHLHB2** | 2.10 | 1.43 | 4.72 | 1.97 |
| **CD44** | 2.10 | 0.95 | 0.72 | 0.78 |
| **APOBEC3F** | 2.09 | 0.90 | 0.61 | 0.81 |
| **EHD1** | 2.09 | 1.05 | 0.71 | 0.83 |
| **C6orf192** | 2.09 | 0.73 | 0.42 | 0.95 |
| **SIK1** | 2.09 | 2.26 | 6.81 | 18.80 |
| **PLK2** | 2.09 | 2.04 | 2.83 | 2.43 |
| **ACSL5** | 2.09 | 0.34 | 0.35 | 0.98 |
| **AKAP12** | 2.09 | 1.13 | 1.34 | 1.37 |
| **BID** | 2.08 | 0.60 | 0.72 | 1.02 |
| **UTX** | 2.08 | 1.57 | 1.82 | 1.56 |
| **NUDCD1** | 2.08 | 1.19 | 0.97 | 1.17 |
| **A4GALT** | 2.08 | 1.15 | 0.97 | 1.87 |
| **ZNF24** | 2.08 | 1.05 | 1.20 | 1.72 |
| **NT5C3** | 2.07 | 1.58 | 2.11 | 1.23 |
| **MKI67IP** | 2.07 | 0.98 | 0.88 | 1.16 |
| **FEM1C** | 2.07 | 1.57 | 1.89 | 1.67 |
| **CHRD** | 2.07 | 1.10 | 0.39 | 1.45 |
| **MTMR11** | 2.07 | 1.06 | 1.74 | 1.04 |
| **BAT5** | 2.07 | 1.44 | 0.90 | 0.91 |
| **DDO** | 2.07 | 1.55 | 1.01 | 1.03 |
| **CD84** | 2.07 | 0.96 | 0.68 | 0.88 |
| **LOC644632** | 2.06 | 1.88 | 0.98 | 2.24 |
| **TMEM149** | 2.06 | 1.45 | 0.88 | 1.50 |
| **MLLT6** | 2.06 | 0.84 | 1.11 | 1.24 |
| **LOC642935** | 2.06 | 2.16 | 1.68 | 2.04 |
| **RP2** | 2.06 | 0.53 | 0.63 | 1.26 |
| **LOC100008589** | 2.06 | 4.65 | 1.29 | 3.81 |
| **GCLM** | 2.06 | 1.74 | 0.75 | 7.51 |
| **SLFN11** | 2.06 | 1.30 | 1.51 | 1.16 |
| **MYO1E** | 2.06 | 1.10 | 1.69 | 1.49 |
| **C1QL2** | 2.05 | 1.70 | 1.63 | 1.43 |
| **LRIG3** | 2.05 | 0.94 | 0.98 | 0.99 |
| **TOP1P2** | 2.05 | 1.19 | 0.74 | 1.96 |
| **LOC652183** | 2.05 | 1.00 | 0.25 | 0.62 |
| **TGM1** | 2.04 | 0.44 | 0.53 | 0.66 |
| **OAF** | 2.04 | 1.10 | 0.39 | 1.20 |
| **HLA-G** | 2.04 | 1.54 | 1.79 | 1.17 |
| **STK10** | 2.04 | 1.24 | 1.21 | 1.09 |
| **MASTL** | 2.03 | 1.53 | 1.79 | 1.07 |
| **DLEC1** | 2.03 | 1.42 | 1.24 | 1.37 |
| **PHF11** | 2.03 | 1.00 | 1.03 | 1.21 |
| **VAPA** | 2.03 | 0.21 | 0.09 | 1.03 |
| **GLIPR1** | 2.03 | 0.34 | 0.68 | 0.97 |
| **STAT3** | 2.02 | 1.52 | 1.38 | 1.51 |
| **LGMN** | 2.02 | 1.27 | 1.22 | 1.05 |
| **ADPRHL2** | 2.01 | 1.22 | 1.88 | 0.94 |
| **KIAA0082** | 2.01 | 2.36 | 1.28 | 1.74 |
| **LY6E** | 2.01 | 0.93 | 0.68 | 1.07 |
| **RBMS1** | 2.01 | 1.12 | 0.93 | 1.69 |
| **TNFRSF10B** | 2.00 | 2.45 | 3.36 | 1.20 |
| **EIF2AK2** | 2.00 | 1.53 | 1.16 | 1.12 |
